# Fine-tuning of the CRWN2-NTL9 module to repress PR1 expression by a viral effector during geminivirus infection

**DOI:** 10.1016/j.fmre.2025.06.003

**Published:** 2025-06-06

**Authors:** Chenlu Su, Yaqin Wang, Fangfang Li, Yuzhen Mei, Xueping Zhou

**Affiliations:** a State Key Laboratory of Rice Biology and Breeding, Institute of Biotechnology, Zhejiang University, Hangzhou 310058, China; b State Key Laboratory for Biology of Plant Diseases and Insect Pests, Institute of Plant Protection, Chinese Academy of Agricultural Sciences, Beijing 100193, China

**Keywords:** Geminivirus, V2 protein, Crowded nuclei, NTL9, Plant immunity

## Abstract

CROWDED NUCLEIs (CRWNs) are nuclear lamina proteins that play an important role in plant immunity. Nevertheless, the molecular mechanism of how CRWNs regulate host immunity and the mechanism by which pathogens manipulate CRWN-mediated immunity regulation is not understood. In this study, we report that CRWN2 negatively regulates anti-geminiviral defense responses through enhancing the function of NAC with transmembrane motif 1-like 9 (NTL9), and the CRWN2-NTL9 module attenuating host defenses is exploited by the geminivirus tomato yellow leaf curl China virus (TYLCCNV). CRWN2 directly interacts with the transcription factor NTL9 and enhances its binding activity on the promoter of the pathogenesis-related gene *PR1,* to negatively regulate the *PR1* expression, thereby modulating host susceptibility to TYLCCNV. Interestingly, the V2 protein encoded by TYLCCNV enhances the interaction between CRWN2 and NTL9 and subsequently increases the complex's affinity for the *PR1* promoter, thus suppressing *PR1* expression and creating an environment suitable for virus infection. Moreover, the interaction between CRWN2 and V2 is conserved between different geminivirus species, indicating that host immunity regulation mediated by the CRWN2-NTL9 complex is co-opted by different geminiviruses.

## Graphical abstract

**Figure:**
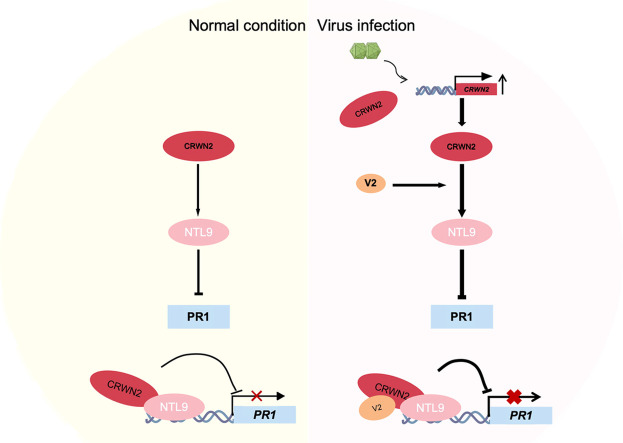

## 1 Introduction

The *Geminiviridae* family contains plant viruses with small, circular, single-stranded genomes, which are encapsidated in twin icosahedral particles and can cause devastating disease in crops [[1], [2], [3]]. Currently, based on host range, insect vector, and genome structure, geminiviruses are classified into 14 genera: *Becurtovirus, Begomovirus, Capulavirus, Citlodavirus, Curtovirus, Eragrovirus, Grablovirus, Maldovirus, Mastrevirus, Mulcrilevirus, Opunvirus, Topilevirus, Topocuvirus,* and *Turncurtovirus* [[4], [5], [6]]. Viruses in the genus *Begomovirus* have either monopartite or bipartite genomes and cause devastating diseases in many economically important crops worldwide [7,8]. Some monopartite begomoviruses were associated with satellite molecules; for example, tomato yellow leaf curl China virus (TYLCCNV) is associated with a betasatellite (TYLCCNB), which acts as a virulence factor [2,[9], [10], [11], [12], [13]].

Owing to the limited coding capacity of the geminivirus genome, viral proteins (such as V2) encoded by geminivirus are often multifunctional effectors. Multiple studies have shown that V2 is a suppressor of gene silencing at both the post-transcriptional level (PTGS) and the transcriptional level (TGS) [14,15]. The geminiviral V2 protein impedes PTGS either by reducing the amplification of the local silencing through competitive SGS3 interaction or by deterring the spread of systemic silencing signal by sequestering vsiRNAs [16,17]. The geminiviral V2 protein directly interacts with NbAGO4 and hinders the interaction of HDA6 with MET1 in the nucleus, which leads to reduced viral DNA methylation and suppression of antiviral TGS [[18], [19], [20]]. Besides the suppression of PTGS and TGS, geminiviral V2 is also essential for viral movement in plants. For example, tomato yellow leaf curl virus (TYLCV) V2 protein plays a crucial role in promoting the nuclear export of the V1 protein and viral systemic infection, likely by transporting TYLCV genomic DNA [21]. In addition, the interaction of C5 and V2 also promotes the nucleoplasm transport of the viral DNA-CP-V2 complex and then guides the viral DNA-protein complex to locate in plasmodesmata along the microfilament, promoting the intercellular movement of the virus [22]. Moreover, V2 also plays a decisive role in viral symptom development [[23], [24], [25]].

The infection cycle of geminiviruses is closely related to the nucleus, such as virus replication and intracellular movement [26]. The eukaryotic nucleus is wrapped in a nuclear envelope, a double-layer membrane structure composed of the outer and inner nuclear membranes [27]. In metazoan cells, the nucleoplasm side of the inner nuclear membrane is covered by an interlaced protein layer called the nuclear lamina, which serves as a key platform for genome regulation due to its proximity to the DNA [28]. The nuclear lamina is vital for various nuclear functions such as providing mechanical support, facilitating signal transduction, organizing chromatin, regulating gene expression, and participating in cell differentiation and metabolism [[29], [30], [31]]. In plants, while lacking direct lamin homologs, nuclear matrix component proteins (NMCPs) serve similar functions. They were initially discovered by analyzing the nuclear matrix structure in carrots [32,33] and were found in most land plants [34]. The carrot NMCPs were identified by analyzing hypothetical proteins exhibiting lamin-like tripartite structures with central coiled-coil domains, and they can organize into the nuclear lamina by forming a filamentous network with coiled-coil domains that localize at the nuclear periphery [35,36]. In *Arabidopsis*, four NMCP homologs known as crowded nuclei (CRWN) proteins, formerly known as LITTLE NUCLEI proteins, have been identified and are widely expressed and involved in maintaining nuclear morphology. Mutations of the *CRWN1* and *CRWN2* genes in *Arabidopsis thaliana* can lead to changes in the shape of the nucleus and make the nucleus smaller [[37], [38], [39]]. In addition, CRWNs are necessary to maintain the H3K27me3 landscape, with tissue-specific chromatin and transcriptional consequences [40]. More specifically, CRWN1 and CRWN4 are crucial for regulating copper tolerance in plants by controlling the expression of copper-associated genes and influencing their positioning at the nuclear periphery under high copper conditions [41]. CRWN3 binds to ABI5 in *A. thaliana*'s nucleus, acting upstream of ABI5 to regulate seed germination by preventing its degradation [42].

In addition to the critical role of CRWNs in plant development, CRWNs also exert important functions in plant immunity. CRWN1 suppresses salicylic acid (SA)-mediated immunity by forming a complex with the NAC transcription factor NTL9 to directly repress the *PR1* gene, and disruption of CRWN function in *crwn1crwn2* double mutant results in hyperactivation of *PR1* and enhanced resistance to the bacterial pathogen *Pseudomonas syringae* [43]. CRWN deficiency (*crwn1crwn2* and *crwn1crwn4*) also triggers spontaneous defense responses through upregulation of the SA biosynthesis gene *Isochorismate Synthase 1* (*ICS1*), leading to premature cell necrosis [44]. Intriguingly, CRWNs exhibit dual regulatory effects on immune signaling. While suppressing SA pathways, CRWN1 simultaneously modulates jasmonic acid (JA) homeostasis and PAMP-triggered immunity (PTI). *crwn1* mutants accumulate elevated JA and JA-Ile levels but display compromised antibacterial resistance due to defective PTI responses, highlighting their role in balancing hormone cross-talk [45]. Meanwhile, several studies have shown that the members of the CRWN family also play important roles in genomic DNA protection. CRWNs contribute to eliminating ROS accumulation, which protects genomic DNA from oxidative damage. CRWNs also physically associate with the DNA damage repair proteins RAD51D and SUPPRESSOR OF NPR1–1 Inducible 1 (SNI1) to maintain genome stability by forming and repairing nuclear bodies at DNA double-strand breaks [46,47]. Whether and how CRWNs regulate plant immunity against viruses remains largely unknown.

The NAC (NAM, ATAF1/2, and CUC2) family is one of the largest families of transcription factors in plants, including 13 members with the transmembrane Motif 1-like (NTL), which have highly conserved NAC domains at the N-terminal and alpha-helical transmembrane (TM) domains at the C-terminal and are classified as membrane-tethered transcription factors [[48], [49], [50], [51]]. NTL9 (NAC with transmembrane motif 1-like 9), also known as CALMODULIN BINDING NAC PROTEIN (CBNAC), is a NAC family transcription factor with a transmembrane domain that plays a key role in a variety of plant biological processes [52]. NTL9 acts as a stress sensor that regulates leaf senescence genes (SAGs) expression through histone modification upon release from the endoplasmic reticulum under osmotic stress [53,54]. NTL9 regulates plant innate immunity by regulating the transcription of defense-related genes involved in the biosynthesis and signaling of salicylic acid (SA), such as *ICS1, EDS1, PAD4, and PR1* [[55], [56], [57]]. Although both CRWNs and NTL9 contribute to plant immunity regulation, the relationship between CRWNs and NTL9 in plant immunity regulation remains elusive, and whether viral effector(s) influence immune responses by CRWNs or NTL9 is largely unknown.

Here, we found that NbCRWN2 enhances the binding capacity of NbNTL9 to the promoter of the *NbPR1* gene, subsequently suppressing the transcription of the *NbPR1* gene and leading to the susceptibility of plants to TYLCCNV. The V2 protein of TYLCCNV interacts with NbCRWN2 and strengthens the interaction of NbCRWN2 with NbNTL9 to inhibit the expression of *NbPR1*. Notably, we found that different geminivirus V2 proteins interact with NbCRWN2 to suppress the transcription of the *NbPR1* gene in a conserved way. Thus, this study reveals a new counter-defense mechanism of fine-tuning the NbCRWN2-NbNTL9 module to repress plant immunity in the nucleus by a viral effector during geminivirus infection.

## 2 Materials and methods

### 2.1 Plant materials, growth conditions, and transgenic plants

Wild-type (WT) and transgenic histone 2B (H2B)-RFP *Nicotiana benthamiana* plants were utilized [58,59]. The experimental plants were cultivated in a greenhouse, and seedlings at an approximately 5-leaf stage were used for the experiments. CRISPR/Cas9-based knockout of *NbCRWN2* in *Nicotiana benthamiana* plants was generated by transformation with the binary vector pBGK01 (Biogle, Changzhou, China) in fusion with single-guide RNA designed to target the ORFs. The synthesized sgRNA oligo was annealed and then ligated to pBGK01 with T4 DNA ligase (Thermo Fisher, Waltham, MA, USA). For overexpression of NbCRWN2 and TYLCCNV V2, the plasmids carrying *2 × 35S::GFP-NbCRWN2* and *2* *×* *35S::TYLCCNV V2*-Falg were used for transformation in *N. benthamiana* plants using standard plant transformation protocols as described previously [60]. qRT-PCR and immunoblot were conducted to verify the gene expression.

### 2.2 Plasmid construction

Full-length ORFs of *NbCRWN2* and *NbNTL9* were amplified from *N. benthamiana* cDNA, synthesized from total RNA using ReverTra Ace qPCR RT Master Mix with gDNA Remover (TOYOBO, Osaka, Japan) following the manufacturer's protocol. To construct plasmids expressing the GFP- or Flag-tagged recombinant proteins, the coding sequences (CDSs) of *NbCRWN2* and *NbNTL9* were individually cloned into binary vectors p35S-GFP or p35S-Flag. For bimolecular fluorescence complementation (BiFC) assays, the CDSs of *NbCRWN2, NbNTL9*, and *TYLCCNV V2* were cloned into p2YN or p2YC, respectively. For TRV-based VIGS, the 200–300-bp fragments of *NbNTL9* and *NbPR1* were constructed into the TRV *RNA2* vector. For electrophoretic mobility shift assay (EMSA) assays, the CDSs of *NbNTL9, NbCRWN2, TYLCCNV V2* and *V2*_*A21P*_ were cloned into pMBP28a or pET28a, respectively. Primer sequences utilized for the cloning are detailed in Table S1.

### 2.3 Agroinfiltration and virus-induced gene silencing

As previously described*, N. benthamiana* plants at the 5- to 7-leaf stage were subjected to agrobacterium infiltration [61]. The VIGS system, based on TRV vectors, was employed to silence *NbNTL9. Agrobacterium tumefaciens* containing pTRV1 was co-infiltrated with either pTRV2-GUS (as a control) or pTRV2-NbNTL9 at a 1:1 ratio. Plants were analyzed 12 days post-infiltration.

### 2.4 Site-directed mutagenesis

Mutants of TYLCCNV V2 were generated using a Site-Directed Mutagenesis Kit (Sangon Biotech Co., Ltd., China). Specifically, an alanine at amino acid position 21 was substituted with proline (GCA to CCA), creating NbCRWN2-interaction-compromised V2 mutants. Primers for mutagenesis are listed in Table S1.

### 2.5 RNA extraction and real-time quantitative PCR

Total RNA was extracted from *N. benthamiana* plants using TRIzol reagent (Invitrogen, Carlsbad, CA, USA), as described previously. Primer pairs specific to targeting the *NbPR1, NbCRWN17, NbCRWN23, NbCRWN13, NbCRWN*2, *NbCRWN02, NbNTL9, TYLCCNV CP*, and *TLCYnV CP* genes were designed by using Primer3Plus (https://www.primer3plus.com). qRT-PCR was performed using the LightCycler 480 (Roche, Rotkreuz, Switzerland) as described previously.

### 2.6 Split-ubiquitin-based membrane yeast two-hybrid assays

The coding sequence of *NbCRWN2* was PCR-amplified from *N. benthamiana* leaf cDNA, then cloned into the pBT3-C vector, which contains the LexA DNA binding domain and VP16 transactivation domain, facilitating the production of a fusion protein with the C-terminal half of ubiquitin (cUb) at the C-terminus of NbCRWN2. The full-length coding sequence of TYLCCNV *V2* or *V2* mutants (*V2_A21P_*) and *NbNTL9* was cloned into the pPR-C vector, expressing the fusion protein with the N-terminus of NubG (nUb). Co-transformations of pBT3-NbCRWN2 with either pPR-V2 or -V2_A21P_ or NbNTL9 were conducted in the *Saccharomyces cerevisiae* strain NMY51 (Dualsystems Biotech, Zurich, Switzerland). Transformants were selected on synthetic -Leu/-Trp medium (72 h, 30 °C), followed by interaction assays on -Leu /-Trp/-His medium with 5 mM 3-AT or -Leu /-Trp/-His/-Ade.

### 2.7 Split-ubiquitin-based membrane yeast three-hybrid assays

The full-length coding sequence of the *NbNTL9* and *TYLCCNV V2* combination or the *NbNTL9* and *TYLCCNV V2* mutant (*V2_A21P_*) combination was cloned into the pPR bridge vector, expressing the nUb-NbNTL9 fusion protein with the N-terminus of NubG (nUb) driven by the *CYC1* promoter and conditionally expressing *TYLCCNV V2* driven by the *MET25* promoter in the absence of methionine. Co-transformations of pBT3-NbCRWN2 with either pPR-NTL9-V2 or -NTL9-V2_A21P_ were conducted in the *Saccharomyces cerevisiae* strain NMY51 (Dualsystems Biotech, Zurich, Switzerland). All transformants were grown on synthetic medium -Leu/-Trp at 30 °C for 72 h, then transferred to the SD-Leu/-Trp/-Met medium and SD-Leu/-Trp/-His/-Met medium.

### 2.8 BiFC assay

4-week-old *N. benthamiana* leaves were co-infiltrated with various BiFC constructs. BiFC assays were performed as described previously [61].

### 2.9 Co-IP assay

Protein extraction from co-infiltrated 4-week-old *N. benthamiana* leaves was carried out using an immunoprecipitation (IP) buffer containing 40 mM Tris·HCl (pH 7.5), 150 mM NaCl, 5 mM MgCl_2_, 2 mM EDTA, 5 mM DTT, 0.5% Triton X-100, 5% glycerol, 2 mM PMSF, and an EDTA-free protease inhibitor cocktail (Roche, Basel, Switzerland). Soluble proteins were separated by centrifugation, followed by immunoprecipitation using Anti-FLAG M2 or Anti-GFP Magnetic Beads (Sigma-Aldrich, MO, USA). The Co-IP assay was then performed as described previously [11].

### 2.10 Yeast growth kinetics

The transformed yeast cultures were grown individually until approximately OD_600_ = 1.0 in SD/-Leu/-Trp medium. The cultures were collected and resuspended by using the lipid SD/-Leu/-Trp/-Met and SD/-Leu/-Trp/-Met/-His medium individually until the approximately OD_600_ = 0.4. The suspensions were transferred to the multi-well plate, and each well was filled with 200 µL of suspension. The multi-well plate was incubated in a microplate reader at 30 °C with constant agitation at 150 rpm for 24 h. The growth curves were constructed by measuring the optical density at 600 nm every 30 s.

### 2.11 Western blotting and antibodies

Total protein was extracted from plant tissues using a protein extraction buffer as previously outlined [62]. Equal amounts of protein were separated on 10% SDS-PAGE gels alongside a pre-stained protein ladder (Thermo Fisher Scientific, #26,617, 10–180 kDa; Shanghai Epizyme Biomedical Technology, #WJ103, 10–250 kDa) and blotted onto NC membranes. The membranes were probed with specific primary antibodies against eGFP (cat: AE012, ABclonal, Wuhan, China), FLAG tag (cat: F1804, Sigma, St. Louis, MO, USA), and actin (cat: AC009, ABclonal, Wuhan, China). Primary antibodies against TYLCCNV/TLCYnV CP were generated in our laboratory.

### 2.12 Yeast protein extraction

Yeast monoclonal was selected from the SD/-Leu/-Trp/-Met plate and inoculated into 2–5 ml of SD/-Leu/-Trp/-Met liquid medium at 250 rpm at 30 °C for 2 days. Cells were harvested by centrifugation at 5000 rpm for 10 min at room temperature, and the supernatant was removed. The pellet was resuspended in 100 µL ddH₂O and mixed, then treated with 100 µL of 0.2 M NaOH to disrupt the cell wall. After incubation at room temperature for 5 min, the cells were centrifuged at 13,000 rpm for 1 min, and the supernatant was removed. After adding 30 µL of 4XSDS-PAGE loading buffer, denature for 5–10 min at 95 °C, centrifuge for 2 min at room temperature at 12,000 rpm, and remove the supernatant for western blot analysis.

### 2.13 Luciferase activity detection assay

Full lengths of *NbNTL9, TYLCCNV V2,* and *TYLCCNV* V2_A21P_ were subcloned to pCAMBIA1391-Flag, and the *NbPR1* promoter was subcloned to pBinplus-luc, and the *NbPR1* promoter without ATG was used as a control. The *NbPR1* promoter-driven luciferase gene was delivered by *GV3101* into tobacco leaves with NbNTL9/Vector, TYLCCNV V2/V2_A21P_, or vector alone via infiltration. Post infiltration (after 48 h), leaves were punched into 96-well plates and allowed to equilibrate at room temperature for 1 hour. Subsequently, 100 mM fluorescein (Sangon Biotech) was injected into the leaves, and they were kept in the dark for 6 min before fluorescence quenching. Luciferase activity was quantified using the Chemiluminescence Imaging Analysis System (Tanon, Shanghai, China).

### 2.14 Electrophoretic mobility shift assay

The recombinant MBP, MBP-NbNTL9, His-Tag, His-NbCRWN2, His-TYLCCNV V2, and His-TYLCCNV V2_A21P_ proteins were purified from the *Escherichia coli* strain BL21. His tag-contained proteins were purified by using the His-Tag Protein Purification Kit (Beyotime, Shanghai, China) and MBP and MBP-NTL9 were purified by using the MBP-Tag Protein Purification Kit (Frdbio, Wuhan, China). The concentration of purified proteins was quantified using a NanoDrop instrument (NanoDrop Technologies, Wilmington, DE, USA). The *NbPR1* promoter's element (TAA TGC TTT AGG TGG TGA AGT ATA G) and its reverse complement were synthesized and 3′ biotinylated by Sangon Biotech. The single-strand DNAs were mixed, denatured at 95 °C for 5 min, and slowly cooled to room temperature to form the double-strand DNAs. EMSA was performed using the Chemiluminescent EMSA Kit (cat: GS009, Beyotime, Shanghai, China) [43].

### 2.15 Data mining and phylogenetic analyses

To identify potential CRWN family members in *N. benthamiana,* CRWN paralogs were retrieved through keyword, BLASTn, and BLASTp searches on the *N. benthamiana* draft genome sequence data (https://solgenomics.net/organism/Nicotiana_benthamiana/genome). For evolutionary analysis, CRWN sequences from *Arabidopsis thaliana* were obtained from NCBI (http://www.ncbi.nlm.nih.gov/). Multiple sequence alignments and maximum likelihood (ML) phylogenetic trees with 1000 bootstrap replicates were constructed using MEGA version 11 software [63].

### 2.16 Alphafold prediction of protein interactions

Amino acid sequences of NbCRWN2 and TYLCCNV V2 were input for protein interaction prediction. Complex models were generated using AlphaFold2-Multimer [64] via the ColabFold platform [65]. Interface residues in the NbCRWN2-TYLCCNV V2 complexes were identified using the “InterfaceResidues” ChimeraX script.

### 2.17 Quantification and statistical analysis

All experiments involving measurements, quantifications, and imaging were performed in triplicate or more. GraphPad Prism version 9 or Excel was employed for data visualization and statistical testing. Statistical parameters such as mean ± standard deviation (SD), standard error (SE), and 95% confidence intervals are indicated in figure legends. Statistical significance is denoted by asterisks in all graphs: *P < 0.05; ^⁎⁎^*P* < 0.01; NS, *P* > 0.05 (not significant), as determined by a two-tailed Student’s *t*-test.

### 2.18 Accession numbers

Genomic locus identifiers for key genes are as follows: *Arabidopsis CRWN1* (AT1G67230.1), *CRWN2* (AT1G13220.2), *CRWN3* (AT1G68790.1), *CRWN4* (AT5G65770.2); *N. benthamiana NbCRWN17* (Niben101Scf00388g08017.1), *NbCRWN23* (Niben101Scf11355g01023.1), *NbCRWN2* (Niben101Scf22781g00001.1), *NbCRWN02* (Niben101Scf03254g03002.1), *NbNTL9* (Niben101Scf00308g01003.1).

## 3 Results and discussion

### 3.1 NbCRWN2 negatively regulates host defenses against TYLCCNV

**Fig. 1:**
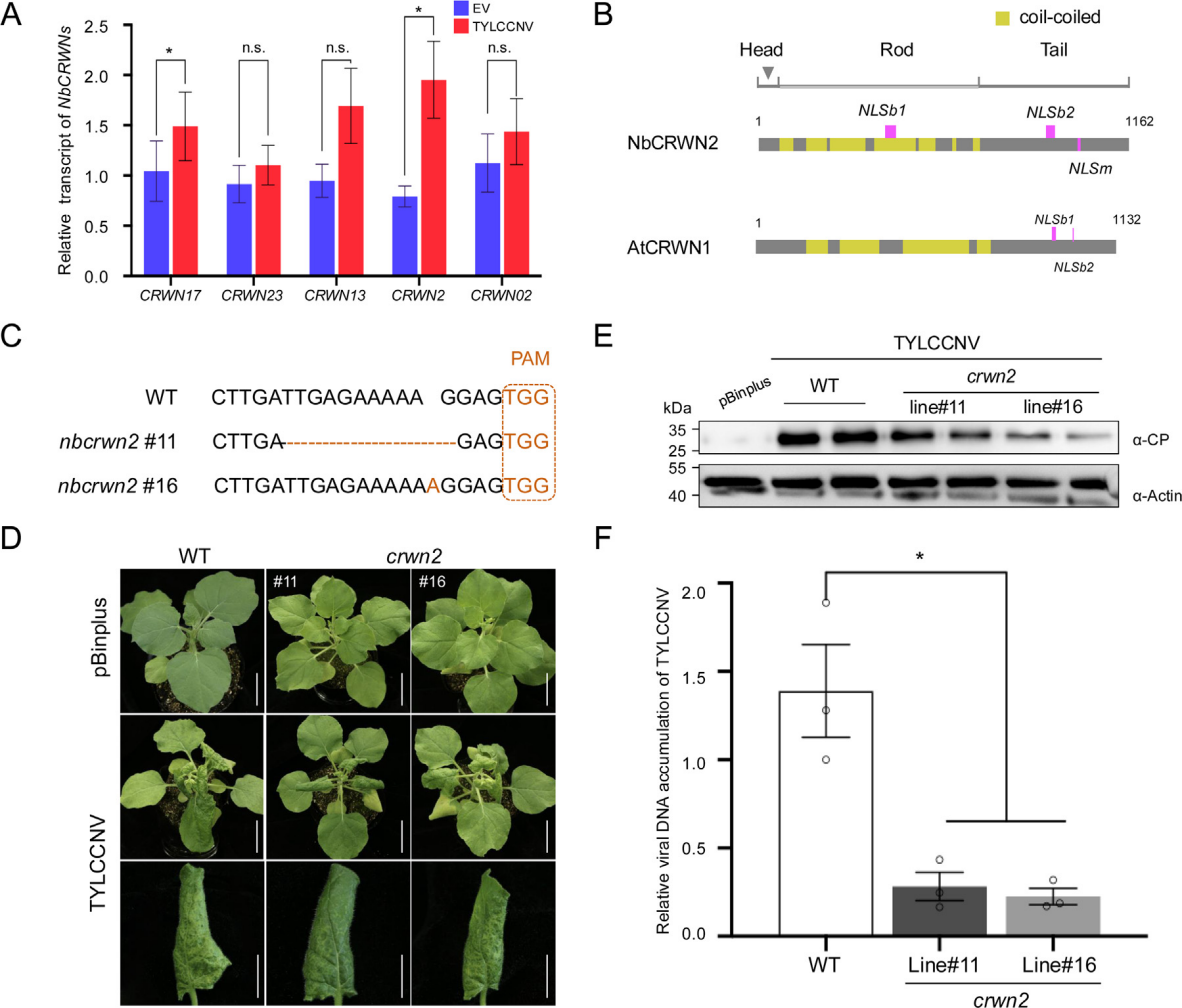
**NbCRWN2 negatively regulates host defenses against TYLCCNV.** (A) The *NbCRWN2* transcript level in mock or TYLCCNV-infected *N. benthamiana* systemic leaves at 10 days post-inoculation (dpi); mock: Agrobacterium carrying empty vector-inoculated *N. benthamiana* plants. Data are means ± SD of three biological replicates. Asterisks mark significant differences according to a two-tailed Student’s *t-*test, *P < 0.05. (B) Schematic representation of NbCRWN2 and its homolog from *Arabidopsis* (AtCRWN1). The NLS motifs are indicated by pink vertical bars and predicted by NLStradamus (http://www.moseslab.csb.utoronto.ca/NLStradamus/). The head, rod, and tail domains are represented by different colors using the MultiCoil program (http://multicoil.lcs.mit.edu/cgi-bin/multicoil). (C) Sanger sequencing to confirm the mutation pattern in *nbcrwn2 N. benthamiana* plants. The PAM sequence of the target sequence is labelled by the orange dashed box. The mutation pattern is annotated by the orange dotted line. (D) Photographs of representative TYLCCNV symptoms in WT and *nbcrwn2 N. benthamiana* plants at 11 dpi. At least 6 WT and *nbcrwn2 N. benthamiana* plants were examined in infection assays. The experiments were repeated at least three times with similar results. Scale bar = 3 cm. (E) Immunoblot analysis of TYLCCNV accumulation in systemic leaves of TYLCCNV-infected WT and *nbcrwn2 N. benthamiana* plants at 11 dpi. Actin was used as a loading control. (F) Quantitative real-time PCR (qPCR) analysis of relative accumulation levels of TYLCCNV in WT and *nbcrwn2 N. benthamiana* plants. Data are means ± SD of three biological replicates (each with four technical replicates). Asterisks mark significant differences according to a two-tailed Student’s *t*-test, *P < 0.05. *Nbactin* was used as an internal reference.

To elucidate whether the *CRWN* genes could be responsive to geminivirus infection, we detected the relative transcripts of *NbCRWNs* in mock and TYLCCNV-infected *N. benthamiana* plants. Quantitative RT-PCR (qRT-PCR) results showed that no significant difference in the expression of *NbCRWN02, NbCRWN13, and NbCRWN23* was detected between mock and TYLCCNV-infected *N. benthamiana* plants. In contrast, the transcripts of *NbCRWN2* and *NbCRWN17* were notably up-regulated in TYLCCNV-infected leaves (Fig. 1A). These results indicate that *NbCRWN2* and *NbCRWN17* are two important genes responsive to TYLCCNV infection. Phylogenetic analysis revealed that NbCRWN2 has a close homologue with AtCRWN1, which was reported as an important factor in plant immunity (Fig. S1A), so we selected NbCRWN2 for further study. The predicted secondary structure of both NbCRWN2 and AtCRWN1 contains a large central coil region (Fig. 1B); in parallel, a long coil-coiled intermediate helix could also be predicted in both NbCRWN2 and AtCRWN1 three-dimensional structures by using Alphafold II software (Fig. S1B). To study whether NbCRWN2 is involved in host resistance against geminivirus, *NbCRWN2* was knocked out using CRISPR-Cas9-mediated genome editing. Sanger sequencing confirmed that the *NbCRWN2* coding sequence was shifted by CRISPR/Cas9-mediated genome editing (Figs. 1C and S2A). We then inoculated WT and T2 generation *nbcrwn2* plants with TYLCCNV. The negative regulatory role of NbCRWN2 in antiviral defense was demonstrated by the observation that *nbcrwn2* plants exhibited milder viral symptoms, lower TYLCCNV CP, and reduced viral genome accumulations compared to wild-type plants (Fig. 1D-F). These results indicate that NbCRWN2 has a negative function in plant resistance against TYLCCNV.

### 3.2 NbNTL9, as an interactor of NbCRWN2, also negatively regulates plant defense against TYLCCNV

**Fig. 2:**
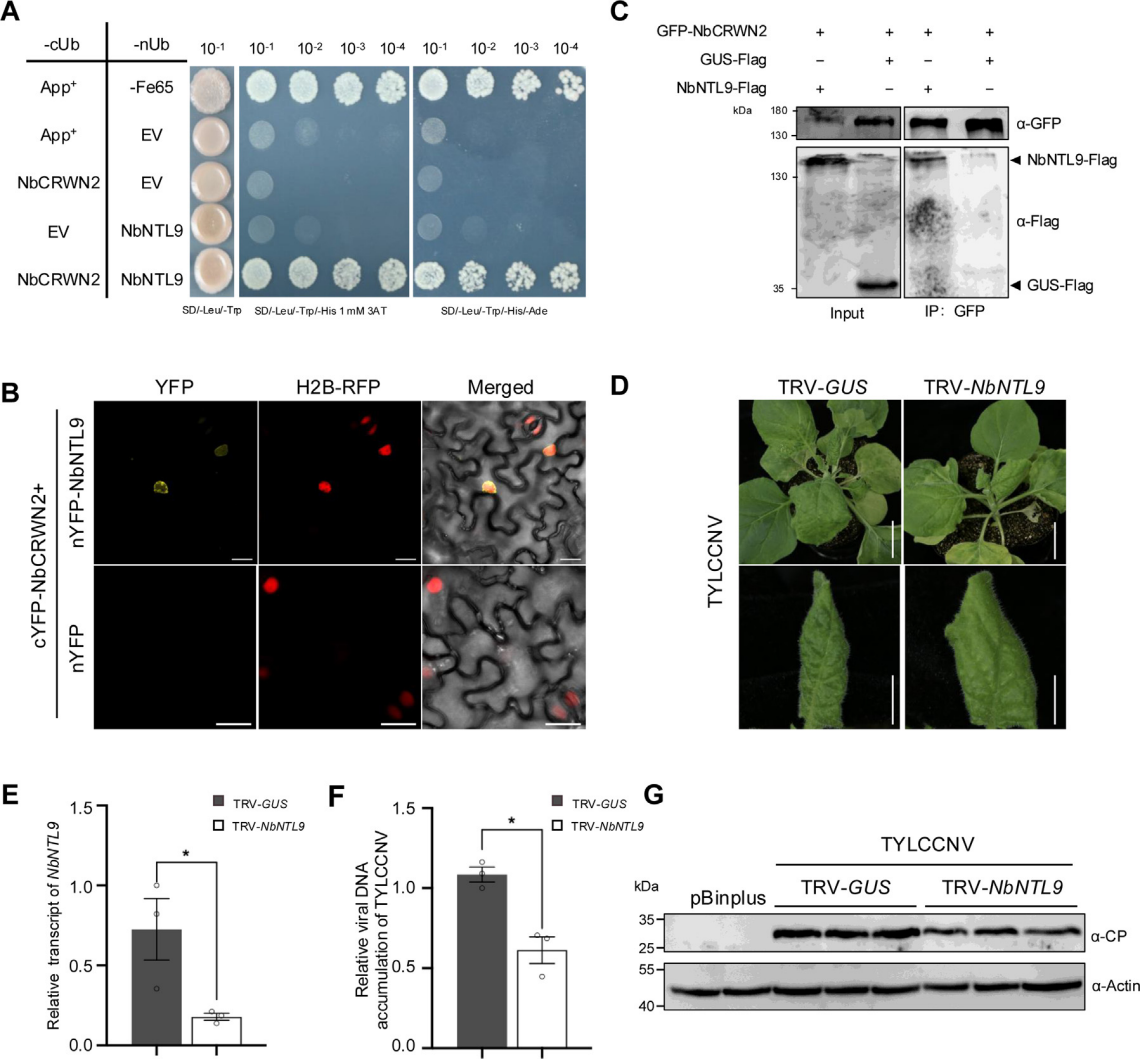
**CRWN2 interacts with NTL9, negatively regulates host defense against TYLCCNV.** (A) Identification of the interaction between NbCRWN2 and NbNTL9 in yeast cells. The NMY51 yeast strain co-transformed with the indicated plasmids was subjected to 10-fold serial dilution and grown on the selective medium SD/-Leu/-Trp/-His supplemented with 1 mM 3-AT and the selective medium SD/-Leu/-Trp/-His/-Ade at 30 °C. (B) Bimolecular fluorescence complementation (BiFC) analysis of NbCRWN2 and NbNTL9 interaction in H2B-RFP transgenic *N. benthamiana* leaves. The nYFP-NbNTL9 and cYFP-NbCRWN2 constructs were co-transformed into H2B-RFP transgenic *N. benthamiana* leaves. nYFP was co-transformed with cYFP-NbCRWN2 to serve as a negative control. Scale bar = 20 µm. (C) Co-immunoprecipitation (Co-IP) assays showing the interaction of NbCRWN2 and NbNTL9 in vivo. GFP-NbCRWN2 was co-expressed with NbNTL9-Flag or GUS-Flag in *N. benthamiana* leaves. Total proteins were extracted and immunoprecipitated with anti-GFP magnetic beads. The proteins were detected with anti-Flag and anti-GFP antibodies. * represents GUS-Flag; ** denotes NbNTL9-Flag. (D) The symptoms of TYLCCNV-infected mock (TRV-*GUS*) and *NbNTL9-silenced* (TRV-*NbNTL9*) *N. benthamiana* plants at 9 dpi. At least 6 mock and *NbNTL9-silenced N. benthamiana* plants were examined for TYLCCNV infection. The experiments were repeated at least three times with similar results. Scale bar = 3 cm. (E) Silencing efficiency of *NbNTL9* detected by qRT-PCR. Data are means ± SD of three biological replicates. Asterisks indicate a statistically significant difference according to the Student’s *t*-test (two-tailed), *P < 0.05. (F) qPCR analysis of the relative DNA accumulation of TYLCCNV in control (TRV-GUS) and *NbNTL9-silenced* (TRV-NbNTL9) *N. benthamiana* plants. Data are means ± SD of three biological replicates. Asterisks indicate a statistically significant difference according to the Student’s *t*-test (two-tailed), *P < 0.05. (G) Immunoblot analysis of TYLCCNV accumulation in systemic leaves of TYLCCNV-infected control (TRV-GUS) and *NbNTL9-silenced* (TRV-NbNTL9) *N. benthamiana* plants at 11 dpi. Actin was used as a loading control.

CRWN proteins have recently been identified as masters in the regulation of defense signaling via recruiting NAC WITH TRANSMEMBRANE MOTIF1LIKE9 (NTL9) in *Arabidopsis thaliana* [43]. To further elucidate the molecular mechanism of the role of NbCRWN2 in plant immunity regulation, we performed yeast two-hybrid (Y2H) assays to identify the interaction between NbCRWN2 and NbNTL9. Y2H results showed that NbCRWN2 interacts with NbNTL9 (Fig. 2A). We next confirmed the interaction in co-immunoprecipitation (Co-IP) and bimolecular fluorescence complementation (BiFC) assays (Fig. 2B-C), which showed that NbCRWN2 interacted with NbNTL9 at the nuclear periphery.

To test whether NbNTL9, identified above, has biological relevance during TYLCCNV infection, we silenced the *NbNTL9* gene in *N. benthamiana* plants by using a tobacco rattle virus (TRV)-based vector. qRT-PCR analysis revealed that the transcript of *NbNTL9* was significantly reduced (over 70%) in TRV-NbNTL9-treated plants compared to mock (TRV-GUS) (Fig. 2E). We inoculated *NbNTL9*-silenced and mock (TRV-*GUS*)-treated plants with TYLCCNV, respectively. Inoculation assays showed that more severe symptoms (such as leaf curling) were easily observed in mock-treated plants at 9 dpi, while *NbNTL9*-silenced plants showed milder symptoms (Fig. 2D). qPCR and western blot analysis validated that the accumulation of viral DNA and coat protein (CP) in *NbNTL9*-silenced plants was significantly lower than that in mock-treated *N. benthamiana* plants (Fig. 2F, G). These findings demonstrate that silencing of *NbNTL9* attenuates viral symptom development, indicating that *NbNTL9* negatively regulates plant resistance against TYLCCNV infection.

### 3.3 NTL9 suppresses the expression of PR1 and functions downstream of CRWN2

**Fig. 3:**
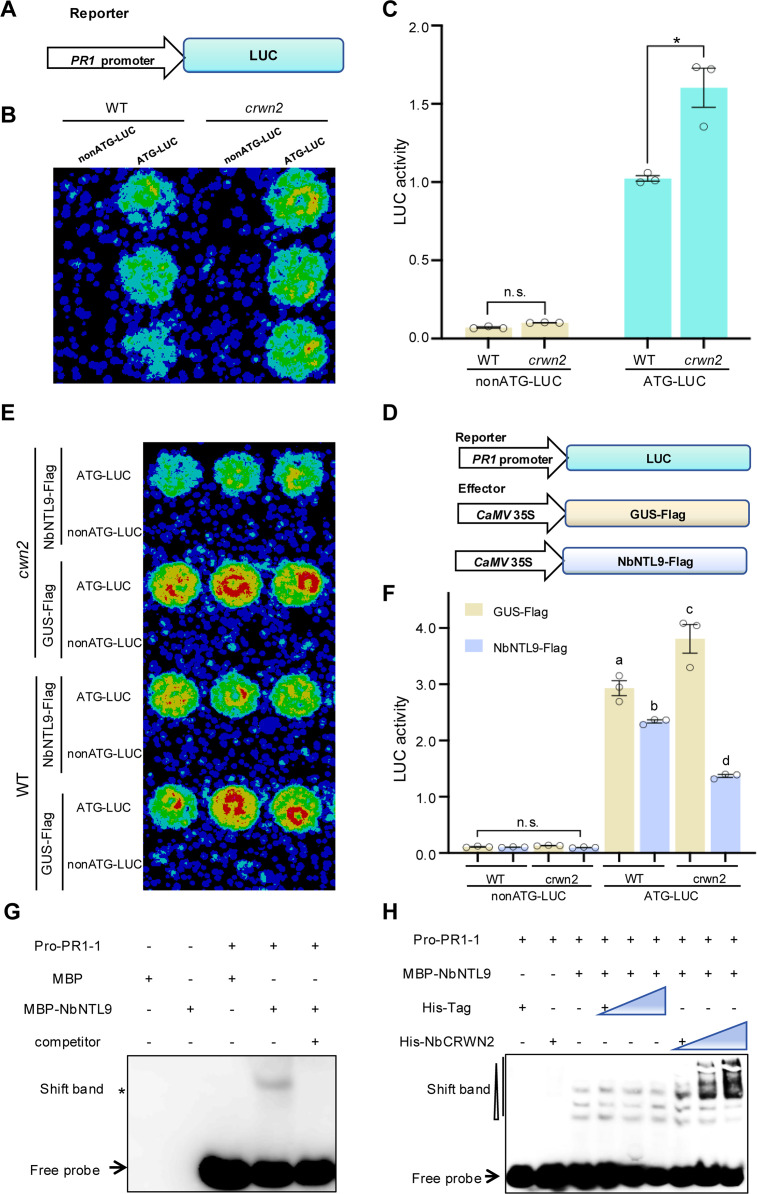
**NTL9 suppresses *PR1* expression and functions downstream of CRWN2.** (A) Schematic of reporter system used in analyzing the transcription level of *NbPR1*. The DNA fragment of the *NbPR1* promoter was fused to the coding sequence of firefly luciferase (LUC). (B) The expression of LUC in WT and *nbcrwn2 N. benthamiana* leaves expressing LUC driven by the *NbPR1* promoter. The construct harboring *NbPR1pro::LUC* was transformed into WT and *nbcrwn2* mutant *N. benthamiana* leaves, and the chemiluminescence was detected at 48 h post-infiltration (hpi). (C) CRWN2 negatively regulates *NbPR1* expression. Bars are the mean ± SD of three replicates (Student’s *t*-test, *P < 0.05; n.s., no significant difference). (D) Schematic of reporter system used in analyzing the effector-mediated expression control of the *NbPR1*, The DNA fragment of *NbPR1* promoter was fused to the coding sequence (CDS) of LUC. The expression of the effector was driven by the CaMV 35S promoter. (E) The expression of LUC in WT and *nbcrwn2 N. benthamiana* leaves expressing LUC driven by the *NbPR1* promoter in the absence or presence of NTL9. The construct harboring *NbPR1pro::LUC* was co-transformed with expression vectors containing *35S::GUS* or *35S::NTL9* into WT and *nbcrwn2 N. benthamiana* leaves, and the chemiluminescence was detected at 48 h post-infiltration (hpi). (F) NTL9 suppresses *NbPR1* expression and functions downstream of CRWN2. Bars are the mean ± SD of three replicates (Student’s *t*-test, *P < 0.05; n.s., no significant difference). Different letters (a, b, c, d) indicate significant differences between groups (*P* < 0.05). (G) NTL9 directly binds to the Pro-PR1–1 element of the *NbPR1* promoter. The non-labeled cold probes were used as competitors to show the binding specificity. Star and arrow point to the positions of shifted bands and free probes, respectively. (H) CRWN2 enhances the binding capacity of NbNTL9 to the Pro-PR1–1 element of the *NbPR1* promoter. Recombinant proteins and biotin-labeled 50 nM Pro-PR1–1 elements were added in all lanes. Lane 1: 1 ug His-Tag; lane 2: 1 ug His-NbCRWN2; lane 3: 1 ug MBP-NTL9; lanes 4–6: 1 ug MBP-NbNTL9 + 1 ug/2ug and 3 ug His-Tag; lanes 7–8: 1 ug MBP-NTL9 + 1 ug/2 ug/3 ug His-NbNCRWN2.

To dissect the mechanism of how NbNTL9 manipulates host resistance and the biological relevance of the interaction between NbCRWN2 and NbNTL9, we constructed a reporter system containing a luciferase reporter gene driven by an *NbPR1* promoter to detect the expression of *NbPR1*, one of the common indicators of *PR* genes, which plays an important role in host defense against pathogens [[66], [67], [68], [69]]. Results of the LUC reporter assays indicated that the luciferase expression was up-regulated in the *nbcrwn2* mutant (Fig. 3A–C). In parallel, the transcript level of *NbPR1* in the *nbcrwn2* mutant was much higher than that in WT plants (Fig. S3A), suggesting NbCRWN2 has an inhibitory effect on *NbPR1* expression. To test the biological role of *NbPR1* during TYLCCNV infection, we performed quantitative PCR to analyze *NbPR1* expression at different time points. Temporal profiling revealed that *NbPR1* transcription in systemic leaves of TYLCCNV-infected *N. benthamiana* plants was significantly increased at early infection stages (10 dpi) but declined at 16 dpi. These results suggest that *NbPR1* is responsive to *TYLCCNV* infection at early infection stages and might play an important role in host defense against TYLCCNV (Fig. S3B). To elucidate the role of NbNTL9 on *NbPR1* expression regulation, we detected the LUC signal in the presence of NbNTL9 and found that NbNTL9 repressed the luciferase expression (Fig. 3D–F). Importantly, we observed that the presence of NbNTL9 in the *nbcrwn2* mutant resulted in a more significant suppression of luciferase expression than that in WT plants (Fig. 3E–F). These results showed that NbNTL9 suppressed the expression of *NbPR1* without the requirement of NbCRWN2, suggesting NbNTL9 exerts its inhibitory functions on *NbPR1* downstream of NbCRWN2.

To explore the molecular mechanism by which the NbCRWN2/NbNTL9 complex interferes with *NbPR1* expression, we performed EMSA to investigate the effects of the NbCRWN2/NbNTL9 complex on *NbPR1* promoter binding. We first predicted the binding region of *NbPR1* using online software (https://plantregmap.gao-lab.org) based on the GCTT core binding region of the *AtPR1* promoter sequence. The potential region of the *NbPR1* bound by NTL9 was obtained (Fig. S3C-D). We next examined the binding capacity of NTL9 on Pro-PR1–1 in EMSA. As shown in Fig. 3G, MBP-NbNTL9 directly binds to the Pro-PR1–1, and the amount of biotin-labeled Pro-PR1–1 bound to NTL9 was decreased upon the addition of unlabeled Pro-PR1–1, impairing NTL9 binding to biotin-labeled Pro-PR1–1. In contrast, neither His-tag nor His-NbCRWN2 binds to the *NbPR1* promoter (Fig. 3G). To further investigate the effects of NbCRWN2 on NTL9 binding capacity on Pro-PR1–1, we introduced NbCRWN2 into EMSA experiments; as the concentration of NbCRWN2 protein increased, more Pro-PR1–1 bound by NTL9 could be detected (Fig. 3H). These results suggest that NbCRWN2 enhances the binding ability of NbNTL9 to the *NbPR1* promoter, thereby enhancing the suppression of *NbPR1* gene expression.

To assess whether NTL9 specifically binds to the promoter of *NbPR1*, chromatin immunoprecipitation-quantitative PCR (ChIP-qPCR) assays were conducted to test NbNTL9 DNA binding ability to promoters of other *PR* genes. For this purpose, we transiently expressed GFP or GFP-NbNTL9 in leaves of *N. benthamiana* plants, then detected the amount of *NbPR1* and *NbPR5* promoters bound by NbNTL9. Interestingly, we found that NbNTL9 specifically binds the promoter of *NbPR1*, but not that of *NbPR5* (Fig. S4). These results suggest that NTL9 specifically binds to the promoter of *NbPR1* for selective regulation of host defense.

### 3.4 Enhanced host defense suppression mediated by the CRWN2/NTL9 complex is co-opted by TYLCCNV V2

**Fig. 4:**
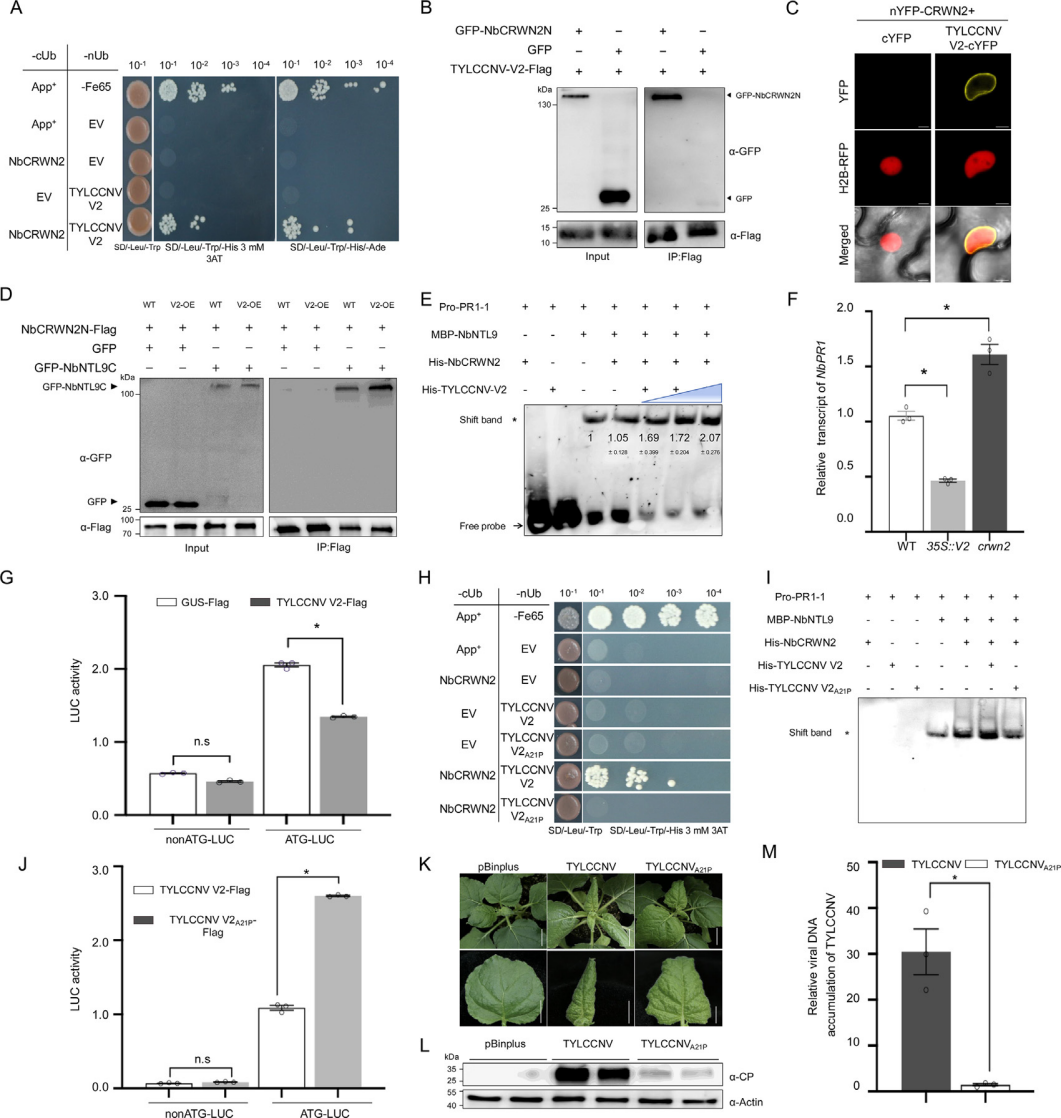
**V2 enhances the plant defense suppression by interacting with NbCRWN2.** (A) Identification of the interaction between NbCRWN2 and TYLCCNV V2 in yeast cells. The yeast (strain NMY51) cells co-transformed with the indicated plasmids were subjected to a 10-fold series dilution and grown on SD/-Leu/-Trp/-His with 3 mM 3AT and SD/-Leu/-Trp/-His/-Ade medium. (B) Co-immunoprecipitation (Co-IP) analysis of the interaction between NbCRWN2N (1–797 aa) and TYLCCNV V2 in vivo. *represents GFP; ^⁎⁎^GFP-NbCRWN2N. (C) Bimolecular fluorescence complementation (BiFC) visualization of the interaction between NbCRWN2 and TYLCCNV V2 in epidermal cells of H2B-RFP transgenic *N. benthamiana* plants. Scale bar = 5 µm. (D) Co-immunoprecipitation (Co-IP) analysis of the interaction between NbCRWN2N (1–797 aa)-Flag and GFP-NbNTL9C (287–631 aa) in WT and *35S::TYLCCNV V2* transgenic *N. benthamiana* plants. V2-OE: *35S::TYLCCNV V2* transgenic *N. benthamiana* plants. (E) TYLCCNV V2 enhances the binding ability of the NbCRWN2-NbNTL9 complex to the Pro-PR1–1 element of the *NbPR1* promoter. Recombinant proteins and biotin-labeled 20 nM Pro-PR1–1 elements were added in all lanes. Lane 1: 1 ug His-NbCRWN2; Lane 2: 1 ug His-V2; Lane 3: 1 ug MBP-NbNTL9; Lane 4: 1 ug MBP-NTL9 + 1 ug His-NbNCRWN2; Lane 5: 1 ug MBP-NTL9 + 2 ug His-NbNCRWN2 + 1 ug His-V2; Lane 6: 1 ug MBP-NTL9 + 2 ug His-NbNCRWN2 + 2 ug His-V2; Lane 7: 1 ug MBP-NTL9 + 2 ug His-NbNCRWN2 + 3 ug His-V2. (F) Quantitative analysis of *NbPR1* transcript level in WT, *35S::TYLCCNV V2,* and *nbcrwn2 N. benthamiana* plants. Data are means ± SD (*n* = 3). Asterisks indicate significant differences according to a two-tailed Student’s *t*-test, *P < 0.05. Data were analyzed using ImageJ software. Values represent mean ± SD (*n* = 3). (G) TYLCCNV V2 suppresses the expression of *NbPR1*. Bars are the mean ± SD of three replicates (Student’s *t*-test, *P < 0.05; n.s., no significant difference). (H) Identification of the key site of TYLCCNV V2 critical for V2-NbCRWN2 interaction in Y2H assays. The yeast (strain NMY51) co-transformed with the indicated plasmids was subjected to a 10-fold series dilution and grown on SD/-Leu/-Trp/-His/ with 3 mM 3AT and SD/-Leu/-Trp/-His/-Ade medium. (I) TYLCCNV V2 enhances the binding ability of the NbCRWN2-NbNTL9 complex to the Pro-PR1–1 element of the *NbPR1* promoter through the direct interaction between V2 and CRWN2. Recombinant proteins and biotin-labeled 20 nM Pro-PR1–1 elements were added in all lanes. Lane 1: 1 ug His-NbCRWN2; Lane 2: 1 ug His-V2; Lane 3: 1 ug His-V2_A21P_; Lane 4: 1 ug MBP-NbNTL9; Lane 5: 1 ug MBP-NTL9 + 2 ug His-NbNCRWN2; Lane 6: 1 ug MBP-NTL9 + 2 ug His-NbNCRWN2 + 1 ug His-V2; Lane 7: 1 ug MBP-NTL9 + 2 ug His-NbNCRWN2 + 2 ug His-V2_A21P_. (J) TYLCCNV V2 suppresses the expression of *NbPR1* through directly interacting with CRWN2. The construct containing *NbPR1pro::LUC* was co-transformed with the expression vectors containing *35S::V2-Flag* and 35S::V2_A21P_-Flag in *N. benthamiana* leaves, and the chemiluminescence was detected at 48 h post-infiltration (hpi). Bars are the mean ± SD of three replicates (Student’s *t*-test, *P < 0.05; n.s., no significant difference). (K) Viral symptoms on *N. benthamiana* plants infected by TYLCCNV or TYLCCNV_A21P_ at 10 dpi. At least 6 *N. benthamiana* plants were examined for each treatment. The experiments were repeated at least three times with similar results. Scale bar = 3 cm. (L) Western blot analysis of virus accumulation in systemic leaves of *N. benthamiana* plants infected with TYLCCNV or TYLCCNV_A21P_. (M) Quantitative PCR analysis of the relative DNA accumulation of TYLCCNV in infected plants. Total DNA was extracted from TYLCCNV or TYLCCNV_A21P_-infected plants to detect the accumulation level of TYLCCNV. *NbActin* was used as a reference gene for normalization. Data were analyzed by Student’s *t*-test, and asterisks denote significant differences between TYLCCNV and TYLCCNV mutant (TYLCCNV_A21P_) (two-sided, *n* = 3, ^⁎⁎^*P* < 0.01).

To better understand the molecular mechanisms underlying the role of CRWN proteins in geminivirus-host interactions, we used yeast two-hybrid screening to discover possible interacting proteins. Initial results demonstrated a specific interaction between TYLCCNV V2 and NbCRWN2. As shown in Fig. 4A, TYLCCNV V2 interacted with NbCRWN2 in yeast cells. We then validated the interaction in BiFC and Co-IP assays, which showed that TYLCCNV V2 also interacted with NbCRWN2 at the periphery of the nucleus (Fig. 4B–C). To test whether the TYLCCNV V2 protein affects the NbCRWN2-NbNTL9 complex formation, NbCRWN2N-Flag and GFP-NbNTL9C were expressed transiently in wild-type and *35S::TYLCCNV V2* transgenic *N. benthamiana* plants. Co-IP results showed that the interaction of NbCRWN2 with NbNTL9 in *35S::TYLCCNV V2* transgenic *N. benthamiana* plants was significantly enhanced (Fig. 4D). Simultaneously, we conducted yeast three-hybrid (Y3H) assays to test the interaction between NbCRWN2 and NbNTL9 in the absence or presence of TYLCCNV V2. Consistent with the above results, the enhanced interaction of CRWN2 with NTL9 could be detected in the presence of TYLCCNV V2 in Y3H assays (Fig. S5). EMSA results showed that the binding capacity of the NbCRWN2-NbNTL9 complex was also enhanced in the presence of TYLCCNV V2 (Fig. 4E). Consistently, the *NbPR1* expression was inhibited in the presence of TYLCCNV V2 in both *35S::TYLCCNV V2* transgenic *N. benthamiana* plants and the LUC report system (Fig. 4F–G), suggesting TYLCCNV V2 might have an inhibitory effect on the *NbPR1* expression. These results indicate that TYLCCNV V2 enhances the binding ability of the NbCRWN2 with the NbNTL9 complex to the *NbPR1* promoter for suppressing *NbPR1* expression.

To delineate the interaction interface of the TYLCCNV V2-NbCRWN2 interaction, we used AlphaFold-Multimer structural prediction software to analyze the structure of the TYLCCNV V2-NbCRWN2 complex (Fig. S6). The structure of the TYLCCNV V2-NbCRWN2 complex showed that the N-terminal α-helix of TYLCCNV V2 is neighboring to NbCRWN2. To analyze the biological relevance of the N-terminal α-helix of TYLCCNV V2, a proline was introduced into the α-helix to break the α-helix structure. And Y2H results confirmed that the N-terminal α-helix of TYLCCNV V2 was critical for TYLCCNV V2-NbCRWN2 interaction since the single-point mutation led to a loss of its interaction (Fig. 4H). Furthermore, we performed Y3H assays to analyze the effects of TYLCCNV V2 and TYLCCNV V2 mutant (TYLCCNV V2_A21P_) on the interaction between NbCRWN2 and NbNTL9. Results of Y3H assays showed that the V2 mutant (V2_A21P_) loses the capacity for enhancing the interaction of NbCRWN2 with NbNTL9 (Fig. S7). To verify that the TYLCCNV V2 mutant (V2_A21P_) identified above has biological relevance, we detected the effects of TYLCCNV V2 and the V2 mutant (V2_A21P_) on the binding capacity of the NbCRWN2-NbNTL9 complex to the *NbPR1* promoter in EMSA assays, different from the TYLCCNV V2 protein, that the V2 mutant (V2_A21P_) loses the ability to promote the binding of the NbCRWN2-NbNTL9 complex to the *NbPR1* promoter (Fig. 4I). As expected, the TYLCCNV V2 mutant (V2_A21P_) was deficient in suppression of the *NbPR1* expression (Fig. 4J). These results indicate that TYLCCNV V2 interferes with the expression of *NbPR1* by enhancing the binding ability of NbCRWN2-NbNTL9 to the *NbPR1* promoter via directly interacting with NbCRWN2.

To investigate the biological relevance of the TYLCCNV V2-NbCRWN2 interaction, we constructed the infectious clone of the TYLCCNV harboring the NbCRWN2-interaction-compromised V2 mutant (V2_A21P_). We then inoculated *N. benthamiana* plants with TYLCCNV and TYLCCNV _A21P_, and symptoms were observed at 10 dpi. In comparison to TYLCCNV-infected plants, those infected with TYLCCNV _A21P_ displayed milder symptoms (Fig. 4K). Additionally, western blot analysis and quantitative PCR (qPCR) revealed lower CP and viral genome accumulation in the TYLCCNV _A21P_-infected plants (Fig. 4L–M). These results suggest that TYLCCNV V2 enhances the *NbPR1* promoter-binding capacity of NTL9 by strengthening the association of the CRWN2/NTL9 complex to suppress the *NbPR1* expression.

### 3.5 CRWN2/NTL9 complex orchestrates broad-spectrum plant resistance against geminiviruses

**Fig. 5:**
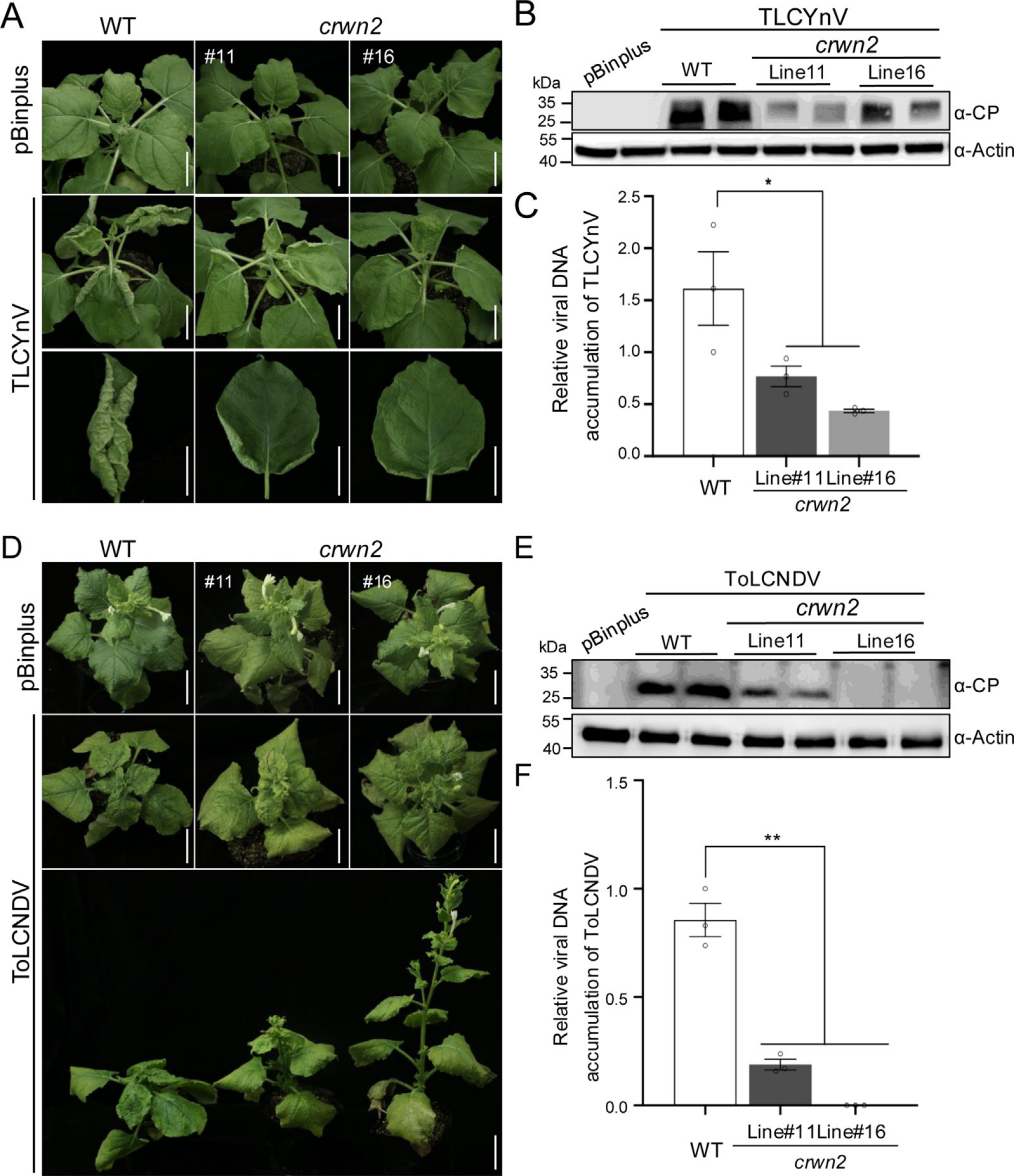
**The CRWN2/NTL9 complex orchestrates broad-spectrum host resistance against geminiviruses.** (A) Viral symptoms in TLCYnV-infected WT and *nbcrwn2 N. benthamiana* plants at 10 days post-inoculation (dpi). At least 6 *N. benthamiana* plants were examined for each treatment. The experiments were repeated at least three times with the similar results. Scale bar = 3 cm. (B) Immunoblot analysis of TYLCYnV accumulation in systemic leaves of TLCYnV-infected WT and *nbcrwn2 N. benthamiana* plants at 10 dpi. Actin was used as a loading control. (C) Quantitative real-time PCR (qPCR) showing the relative DNA accumulation levels of TLCYnV in infected WT and *nbcrwn2 N. benthamiana* plants. Error bars represent the standard deviation of three biological replicates. Statistical analyses were performed using the Student’s *t*-test. *P < 0.05. (D) Viral symptoms induced by ToLCNDV in WT and *nbcrwn2 N. benthamiana* plants at 10 days post-inoculation (dpi). At least 6 *N. benthamiana* plants were examined for each treatment. The experiments were repeated at least three times with the similar results. Scale bar = 3 cm. (E) Immunoblot analysis of ToLCNDV accumulation in systemic leaves of ToLCNDV-infected WT and *nbcrwn2 N. benthamiana* plants at 10 dpi. Actin was used as a loading control. (F) Quantitative real-time PCR (qPCR) showing the relative DNA accumulation levels of ToLCNDV in infected WT and *nbcrwn2 N. benthamiana* plants. Error bars represent the standard deviation of three biological replicates. Statistical analyses were performed using the Student’s *t*-test. *P < 0.05.

To assess if the CRWN2-NTL9 module-involved plant resistance is conserved in other geminiviruses, we inoculated the WT and *nbcrwn2 N. benthamiana* plants with tomato leaf curl Yunnan virus (TLCYnV). Infection assays revealed that the *nbcrwn2 N. benthamiana* plants exhibited milder symptoms, lower CP and viral genome accumulation (Fig. 5A–C). Owing to the similar structure of TLCYnV V2 with TYLCCNV V2, we also detected the interaction between NbCRWN2 and TLCYnV V2 in yeast (Fig. S8). These results suggest that the pathway involved in the NbCRWN2-NbNTL9 complex inhibiting the *NbPR1* expression might be co-opted by different geminivirus species to create a suitable environment for virus infection. To further test whether host resistance mediated by the CRWN2-NTL9 module has potential in breeding broad-resistant crops. WT and *nbcrwn2 N. benthamiana* plants were inoculated with the bipartite geminivirus-tomato leaf curl New Delhi virus (ToLCNDV). Results of infection assays showed that *nbcrwn2 N. benthamiana* plants displayed excellent resistance against ToLCNDV with milder symptoms and lower viral accumulation (Fig. 5D–F). These results indicate that the CRWN2-NTL9 module plays an important role in host defenses and has great potential in breeding resistant crops.

## 4 Discussion and conclusion

CRWNs are plant-specific nuclear matrix constituent proteins with coiled-coil domains essential for maintaining nuclear morphology, organizing heterochromatin, and regulating gene expression. Several studies have reported that *crwn* mutants are hypersensitive to several plant hormones, such as the treatment of SA, JA, or abscisic acid [42,44,45], and multiple abiotic stimuli, including copper stress [41], drought stress [70], and heat stress [71]. The CRWNs in plants are crucial for immunity, and *crwns* knock-out mutant enhances disease resistance against plant pathogens such as bacteria [43,44]. In this study, TYLCCNV infection significantly up-regulated the transcription of *NbCRWN2*, and the *nbcrwn2* mutant displayed elevated resistance against TYLCCNV, which was concomitant with the up-regulation of *NbPR1* expression (Fig. 4). These results indicate that NbCRWN2 negatively regulates plant resistance against TYLCCNV by repressing the high expression of the plant defense gene, *NbPR1*. Further efforts to identify the effects of *crwn2* on key agricultural traits will provide new insights into breeding disease-resistant crops.

Emerging evidence has redefined the role of *PR1* beyond a mere marker of salicylic acid (SA) signaling. Recent studies revealed that ClPR1 directly inhibits zucchini yellow mosaic virus (ZYMV) replication by binding to viral RNA, thereby restricting systemic movement [72]. This functional shift, from a passive indicator to an active antiviral effector, strongly supports the idea that PR1-mediated defenses play an important role in host antiviral immunity.

NTL9, a membrane-anchored NAC transcription factor, is activated via proteolytic processes, either ubiquitin/proteasome-mediated degradation or intramembrane cleavage [73,74]. Considering the direct binding of NTL9 to the *NbPR1* promoter and negatively regulating the *NbPR1* transcription by NTL9, we speculate that NbNTL9 might also dissociate from the membrane to regulate gene expression. This mechanism parallels pathogen-induced activation of membrane-tethered immune regulators in animals (e.g., SREBP cleavage during bacterial infection) [75], highlighting convergent strategies across kingdoms to sense biotic threats at membrane-nucleus interfaces.

The geminivirus V2 protein plays a significant role in the virus infection cycle, which is closely related to the nucleus. V2 mediates the nuclear export of the virus through interacting with exportin-α, which is crucial for viral systemic infection [21]. Our study found that NbCRWN2, a lamin-like protein negatively regulating the expression of the pathogenesis-related gene *NbPR1* by interacting with NbNTL9, is a novel interactor of TYLCCNV V2 (Fig. 4), and TYLCCNV V2 promotes the binding of the NbCRWN2-NbNTL9 complex to the *NbPR1* promoter for suppression of NbPR1 expression. Importantly, we pinpointed the key residue of TYLCCNV V2 involved in the interaction and found that the NbCRWN2-interaction-compromised TYLCCNV V2 mutant (V2_A21P_) was deficient in enhancing the suppression of *NbPR1* expression. These results strongly support the idea that geminivirus co-opts host immunity regulation through the direct interaction between V2 and NbCRWN2. Moreover, the V2 proteins encoded by different geminiviruses also interacted with NbCRWN2 and impeded plant defense responses involved in the CRWN2-NTL9 module, highlighting that while CRWN2-NTL9 confers broad resistance against diverse geminiviruses (e.g., TolCNDV and TLCYnV), viral genetic variability may facilitate evolutionary adaptations to circumvent this defense mechanism, thereby shaping an ongoing arms race between host immunity and pathogen evolution.

**Fig. 6:**
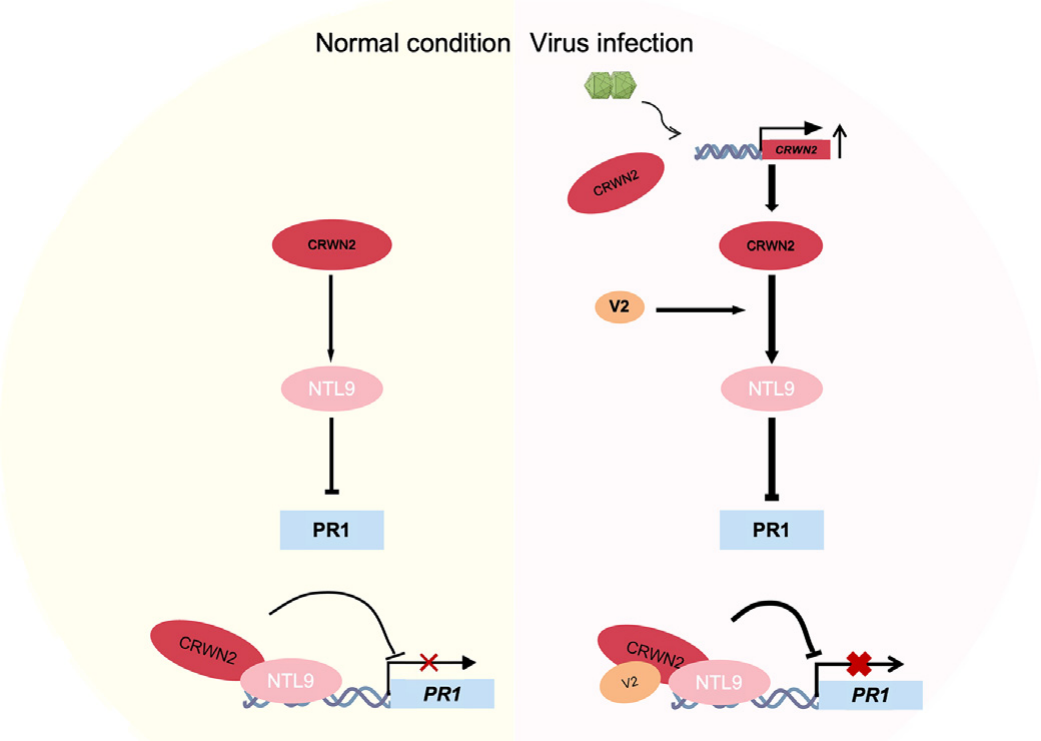
**A proposed working model showing how the geminivirus V2 protein represses PR1 expression by co-opting the NbCRWN2-NbNTL9 module.** Geminivirus infection induces the expression of the *NbCRWN2* gene, which interacts with NbNTL9 to form a complex for binding to the *NbPR1* promoter and repressing the expression of *NbPR1*. The V2 protein encoded by geminivirus enhances the interaction between NbCRWN2 and NbNTL9, leading to an increased suppression of the expression of the *NbPR1* gene. Therefore, the NbCRWN2-NbNTL9 module-mediated suppression of the *NbPR1* gene is co-opted and enhanced by geminivirus V2, which facilitates geminivirus infection.

In summary, we propose a model in which NbCRWN2 interacts with NbNTL9 to form a complex that binds to the *NbPR1* promoter, thereby inhibiting the expression of *NbPR1*. Upon infection by geminivirus, the expression of the *NbCRWN2* gene is upregulated, and the geminiviral V2 protein enhances the interaction of NbCRWN2 with NTL9, leading to a stronger inhibition of the expression of the *PR1* gene (Fig. 6). This model suggests a mechanism by which the virus manipulates host immunity regulators to interfere with defense-related gene expression, potentially as a strategy to facilitate its infection in host plants.

## CRediT authorship contribution statement

**Chenlu Su:** Writing – original draft, Validation, Methodology, Investigation, Formal analysis. **Yaqin Wang:** Methodology, Investigation. **Fangfang Li:** Writing – review & editing, Formal analysis. **Yuzhen Mei:** Writing – review & editing, Writing – original draft, Visualization, Validation, Methodology, Data curation. **Xueping Zhou:** Writing – review & editing, Writing – original draft, Supervision, Project administration, Funding acquisition, Conceptualization.

## Declaration of competing interest

The authors declare that they have no conflicts of interest in this work.

## Appendix Supplementary materials

Image, application 1
