# Supplementary material for: Fine-tuning of the CRWN2-NTL9 module to repress PR1 expression by a viral effector during geminivirus infection
Source: Fundam Res. 2025 Jun 6;6(4):2250–61. doi: 10.1016/j.fmre.2025.06.003 (PMC13424409; doi:10.1016/j.fmre.2025.06.003)
Supplement: Supplementary file 1 [file mmc1.pdf]

Supplementary Information

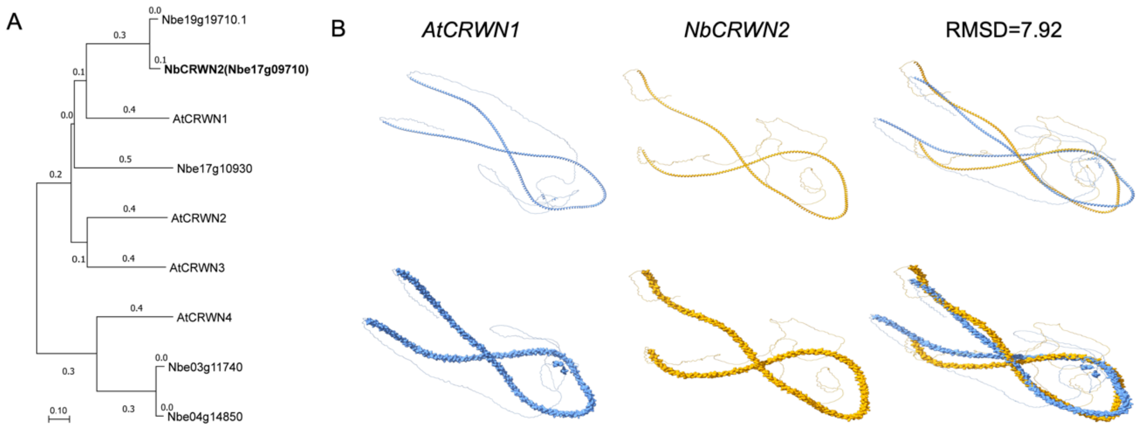

Figure S1. Phylogenetic analysis and three-dimensional protein structures of CRWNs. (A) Phylogenetic analysis of *CRWNs* that were upregulated during TYLCCNV infection with their homologues from Arabidopsis thaliana based on the amino acid sequence using maximum likelihood (ML) phylogenetic trees methods from MEGA 11 software. Bootstrap values (of 1000 replicates) are indicated on each branch. (B) Protein structure prediction using Alphafold II software. The coiled area in the middle of the protein was represented by blue and yellow balls. Root mean square deviation (RMSD) is displayed, which is computed between aligned pairs of the backbone C-alpha atoms in superposed structures in Å. The value below 10 is considered to be structurally similar.

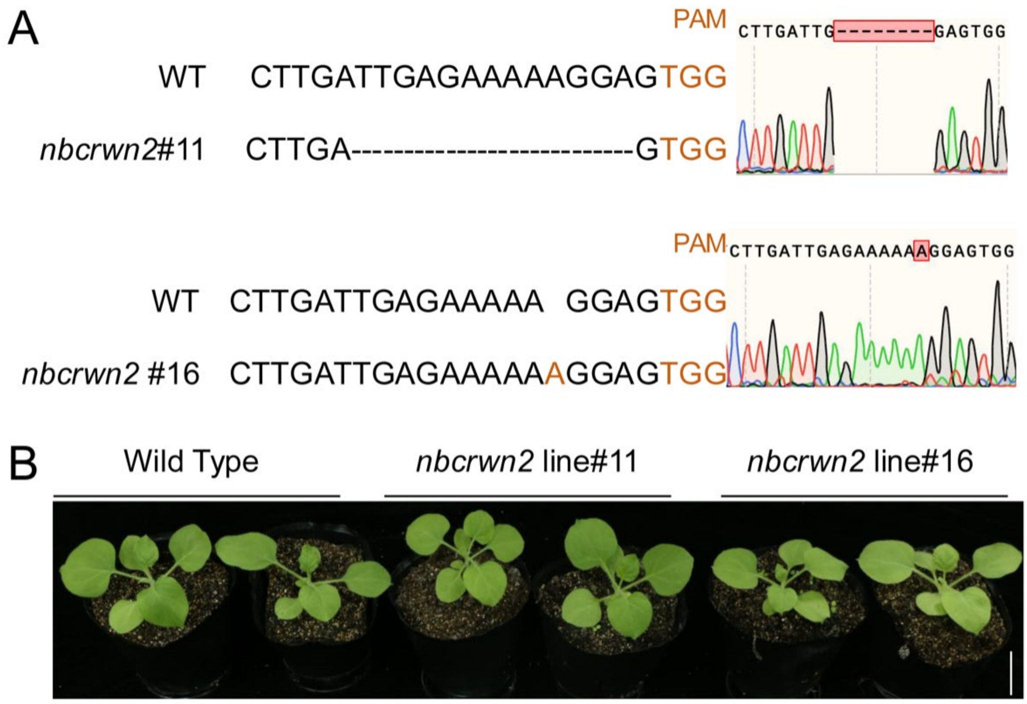

Figure S2. Phenotype of WT and *nbcrwn2 Nicotiana benthamiana* plants. (A) Mutation pattern of *nbcrwn2 N. benthamiana* plants detected by Sanger sequencing. The PAM sequence of the target sequence is labeled by the orange font. The mutation pattern is annotated behind the sequence. (B) Phenotypes of WT and *nbcrwn2 N. benthamiana* plants. Two independent nbcrwn2 lines are shown and the photos were taken at 28 days post-germination. Scale bar = 3 cm.

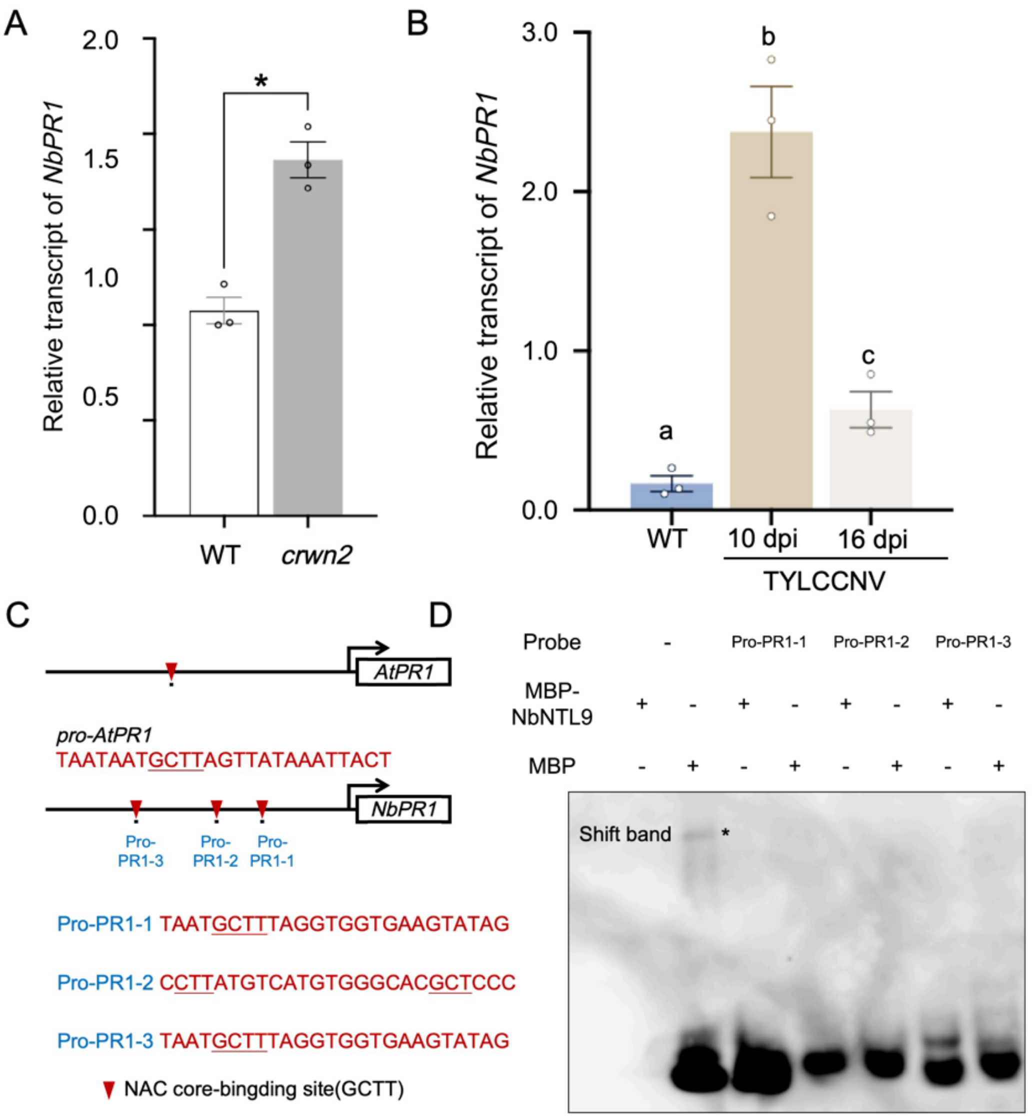

Figure S3. Mapping the NbNTL9-binding sites on the *PR1* promoter. (A) qRT-PCR analysis of the *NbPR1* transcript level in WT and *nbcrwn2 N. benthamiana* plants. Data are means ± SD (n = 3). Asterisks indicate significant differences according to a two-tailed Student's *t*-test, *P < 0.05. (B) Relative transcript levels of *NbPR1* in wild-type at 0, 10 and 16 days post-inoculation (dpi). Bars are the mean ± SEM of three replicates (Student's t-test, *$P < 0.05$; n.s., no significant difference). Different letters (a, b, c, d) indicate significant differences between groups ($P < 0.05$) (C) Diagram of DNA

oligonucleotides used in EMSA. The red triangle represents the GCTT core elements. Pro-PR1-1 represents the *NbPR1* promoter that is most similar to the *AtPR1* promoter, and Pro-PR1-2 and Pro-PR1-3 are the two highest-scoring promoters predicted by the Plant Transcriptional Regulatory Map (https://plantregmap.gao-lab.org). (D) NbNTL9 possesses the capacity of binding to the Pro-PR1-1 element of the *NbPR1* promoter but not to Pro-PR1-2 and Pro-PR1-3. Star shows the positions of shifted bands. * indicates the shift band of Pro-PR1-1 bound by NbNTL9.

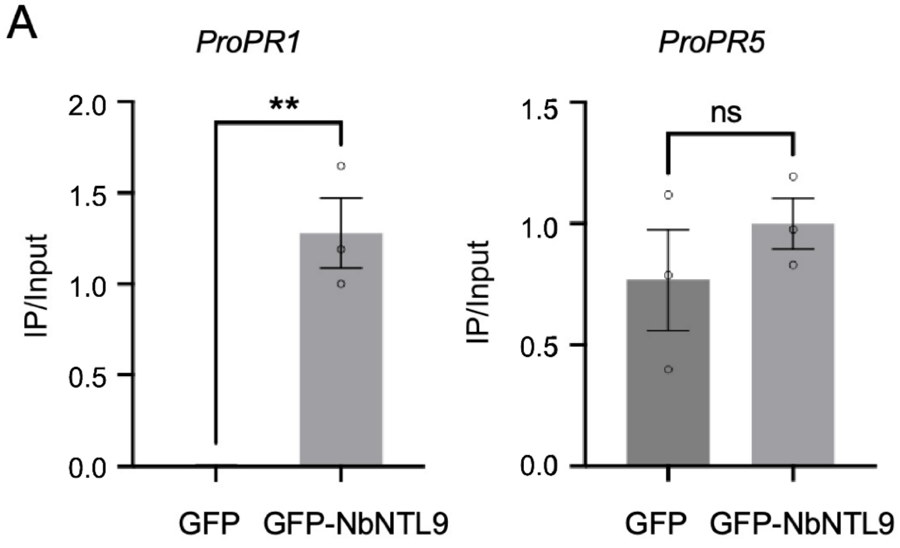

Figure S4. NTL9 specifically binds to the promoter of *NbPR1*. (A) ChIP-qPCR analysis of NbNTL9 binding to *NbPR1* and *NbPR5* promoters in *N. benthamiana* leaves transiently expressing GFP (control) or GFP-tagged NbNTL9 (GFP-NbNTL9) using a GFP antibody. ACTIN was used as internal controls for the ChIP experiments. ChIP-PCR results were quantified by normalization of GFP-IP signal with the corresponding input signal (IP[PR1/ACTIN]/input[PR1/ACTIN]). Data are presented as mean ± SEM (n = 3 biological repeats). Statistical significance was analyzed by one-way ANOVA followed by Tukey’s multiple comparisons test. $^{**}P < 0.01$.

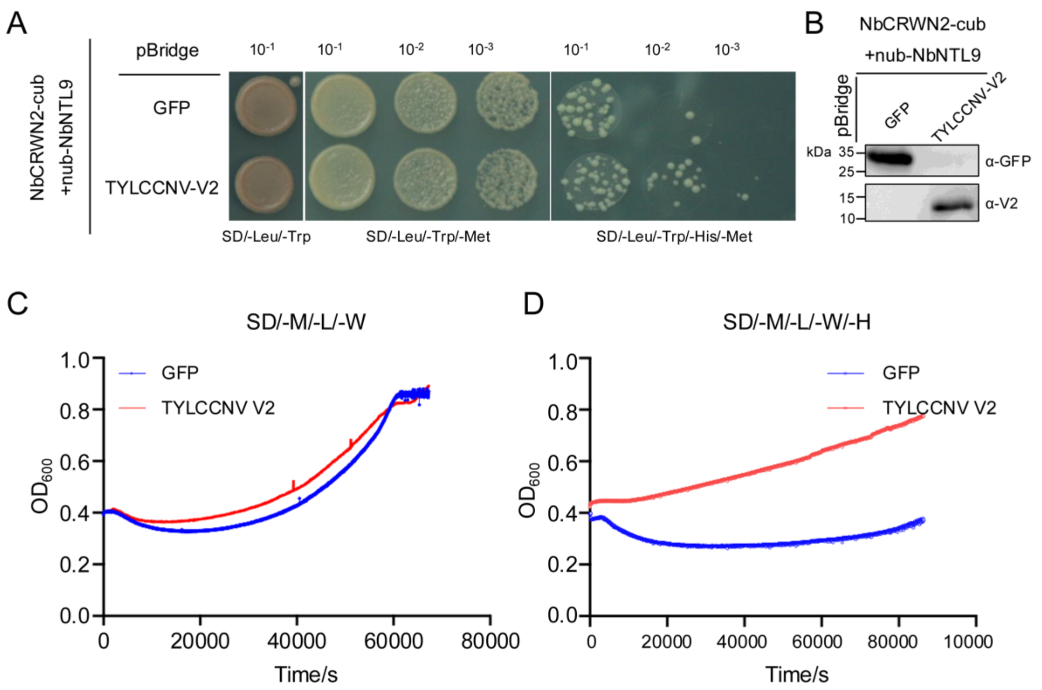

Figure S5. Yeast three-hybrid (Y3H) analysis of the effects of V2 protein on the interaction between NbCRWN2 and NbNTL9. (A) The interaction of NbCRWN2 and NbNTL9 was enhanced in the presence of V2 in Y3H. Yeast strain NMY51 co-transformed with the indicated plasmids were subjected to 10-fold serial dilution and grown on SD/-Leu/-Trp, SD/-Leu/-Trp/-Met, and SD/-Leu/-Trp/-His/-Met medium. (B) Immunoblot analysis of GFP and TYLCCNV-V2 expression in yeast cells of Y3H.The yeast (strain NMY51) co-transformed with the indicated plasmids was cultured in SD/-Leu/-Trp/-Met liquid medium at 30°C for 2 days and was harvested for yeast total protein extraction. GFP and TYLCCNV V2 were detected using GFP and V2 specific polyclonal antibodies individually. (C-D) The growth curve of yeast strain NMY51 co-transformed with the indicated plasmids in SD/-Leu/-Trp/-Met (C) and SD/-Leu/-Trp/-His/-Met medium (D) based on visible absorption. Growth curves were constructed by measuring the optical density at 600 nm every 30 s. Data are presented as the mean of three biological treatments. The blue curve indicates the growth of yeast cells

expressing CRWN2-cUb, nUb-NTL9, and GFP; the red curve indicates the growth of yeast cells expressing CRWN2-cUb, nUb-NTL9, and TYLCCNV V2.

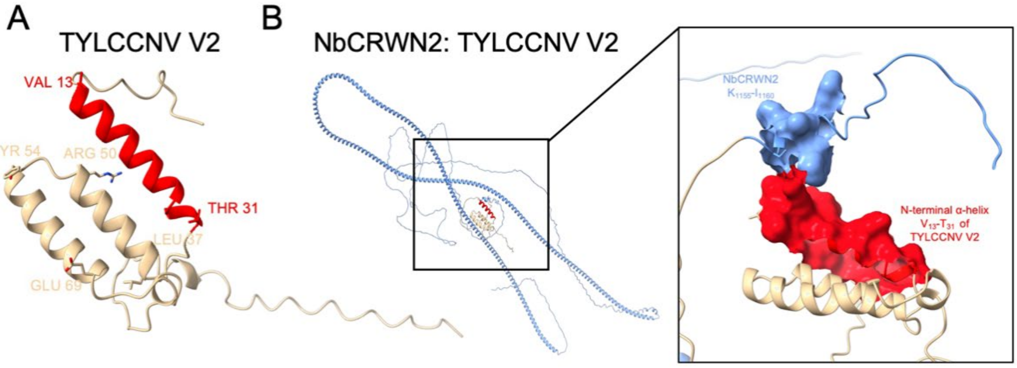

Figure S6. The α-helix at the N-terminus of TYLCCNV V2 is neighboring with NbCRWN2 in the V2/CRWN2 complex predicted by Alphafold. (A) The predicted structure of TYLCCNV V2 from AlphaFold II software, and the N-terminal α-helices first helix was marked red. Three α-helices could be predicted in TYLCCBV V2. Three discontinuous α-helixes were predicted in TYLCCNV V2 at positions V_{13}-T_{31}, R_{37}-L_{50}, and Y_{54}-E_{69}. (B) AlphaFold II structural modeling of the NbCRWN2-TYLCCNV V2 interaction interface. The potential interaction region between NbCRWN2-TYLCCNV V2 was zoomed in the right panel, where the first α-helix in V2, positioned in V_{13}-T_{31}, is closest to NbCRWN2.

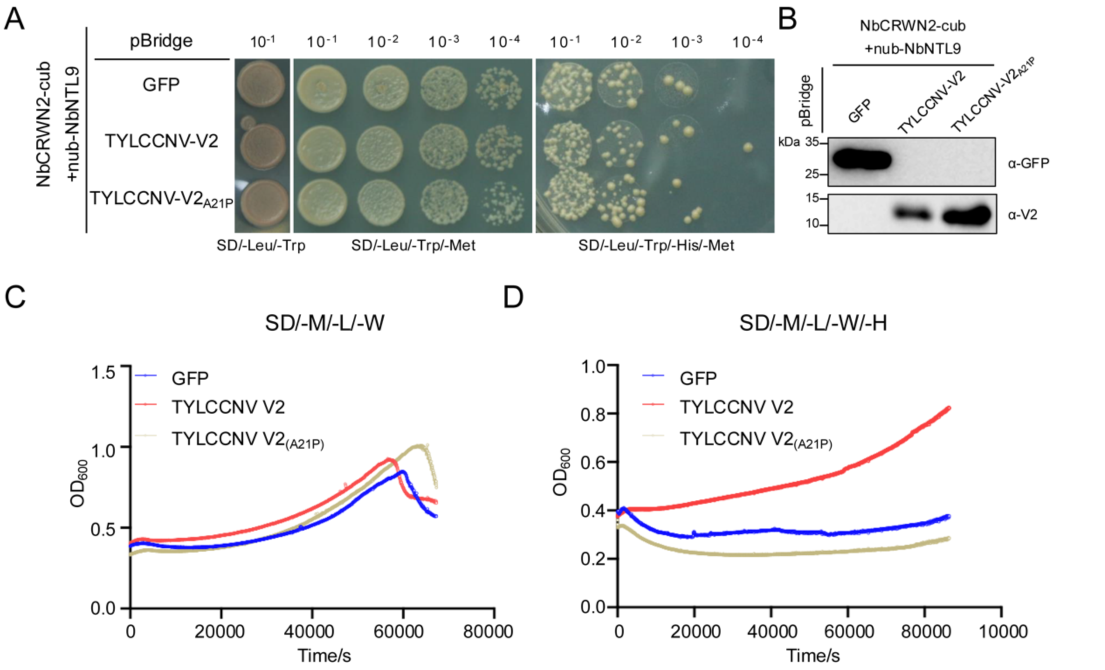

Figure S7. TYLCCNV V2 enhances the interaction between NbCRWN2 and NbNTL9 through its direct interaction with NbCRWN2. (A) Yeast three-hybrid (Y3H) analysis of the effects of interaction between TYLCCNV V2 and NbCRWN2 on NbCRWN2-NbNTL9 complex formation. The yeast (strain NMY51) co-transformed with the indicated plasmids were subjected to 10-fold series dilution and grown on SD/-Leu/-Trp/-Met and SD/-Leu/-Trp/-His/-Met medium. (B) Immunoblot analysis of GFP and TYLCCNV-V2/ $V2_{A21P}$ expression in yeast cells of Y3H. The yeast (strain NMY51) co-transformed with the indicated plasmids was cultured in SD/-Leu/-Trp/-Met liquid medium at 30°C for 2 days and was harvested for yeast total protein extraction. GFP, TYLCCNV V2 and V2 mutant were detected using GFP and V2 specific polyclonal antibodies individually. (C-D) The growth curve of yeast strain NMY51 co-transformed with the indicated plasmids in SD/-Leu/-Trp/-Met (C) and SD/-Leu/-Trp/-His/-Met medium (D) based on visible absorption. Growth curves were constructed by measuring

the optical density at 600 nm every 30 s. Data are presented as the mean of three biological treatments. The blue curve indicates the growth of yeast cells expressing CRWN2-cUb, nUb-NTL9, and GFP; the red curve indicates the growth of yeast cells expressing CRWN2-cUb, nUb-NTL9, and TYLCCNV V2; the brown curve indicates the growth of yeast cells expressing CRWN2-cUb, nUb-NTL9, and TYLCCNV $V2_{A21P}$.

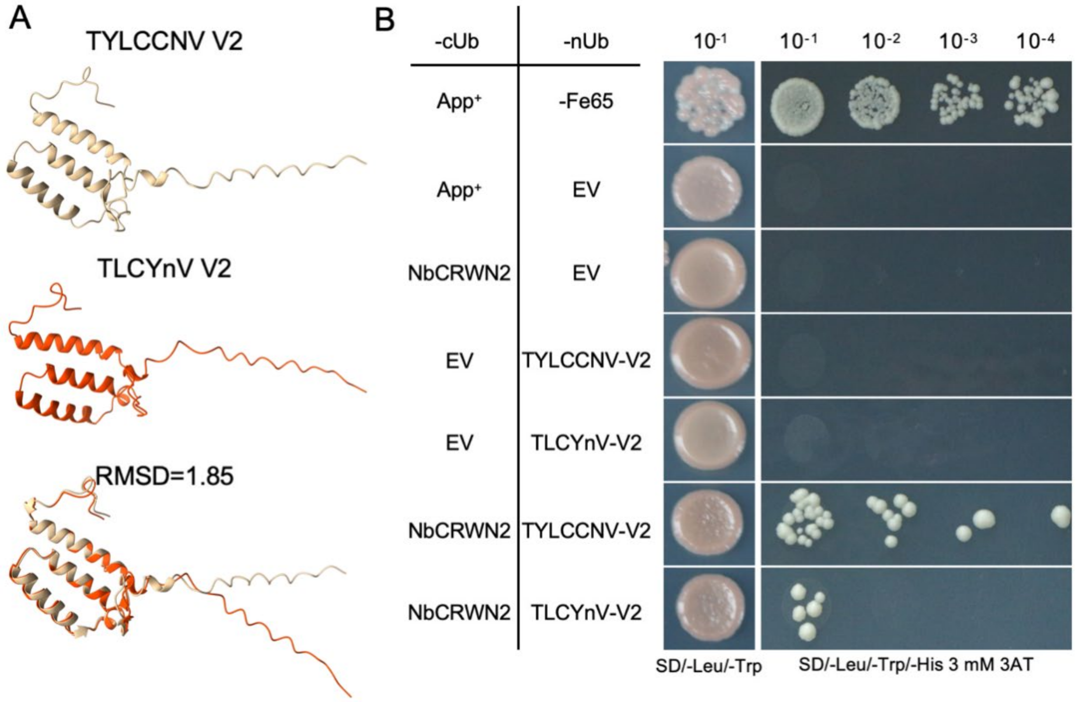

Figure S8. Identification of the interaction between NbCRWN2 and V2 encoded by tomato leaf curl Yunnan virus (TLCYnV). (A) Structural data from AlphaFold II software were used to predict the structure of TYLCCNV V2 and TLCYnV V2, individually. Root mean square deviation (RMSD) is displayed above, which is computed between aligned pairs of the backbone C-alpha atoms in superposed structures in Å. The value below 10 is considered to be structurally similar. Three α-helices could be predicted in TLCYnV V2, which was similar to TYLCCNV V2. Three discontinuous α-helices were predicted in TLCYnV V2 at positions V_{13}-T_{31}, L_{37}-L_{50}, and Y_{54}-G_{70}. (B) Yeast two-hybrid (Y2H) analysis of the interaction between NbCRWN2 and the TLCYnV V2 protein. The yeast (strain NMY51) cells co-transformed with the indicated plasmids were subjected to a 10-fold series dilution and grown on SD/-Leu/-Trp/-His with 3 mM 3-AT medium.

Table S1 Primers used in this study

Primers	Sequences (5’-3’)
**For cloning**	
pBT3c-NbCRWN2-cub-F	CTAATCTAGACTGCAGATGTCTACTCCGCCGAG
pBT3c-NbCRWN2-cub-R	CCCCGACATACTAGTGCTCTGGCAAAGATCCATTTTC
pPRn-nub-TYLCCNV-V2-F	GATTACGCTGGATCCATGTGGGATCCTCTGCTC
pPRn-nub-TYLCCNV-V2-R	GATGCTAGCCCATGGTTAGGGCTTCTGTACATCCG
pPR3n-NbNTL9F-F-BamH1	GATTACGCTGGATCCATGATGGCCGTACTTCCTG
pPR3n-NbNTL9F-R-Nco1	CTAGCCCATGGTTACTACACACACAGTCTAAAGCATTC
pPR3n-TLCYnV-V2-F-BamH1	GATTACGCTGGATCCATGTGGGATCCTTTACTCAACG
pPR3n-TLCYnV-V2-R-Nco1	GATGCTAGCCCATGGTCAGGGCTTCTGTACATCCG
pGD-GFP-CRWN2-F-BamH1	CGGGGATCCATGTCTACTCCGCCGAGAAAGATTTTC
pGD-GFP-CRWN2F-R-Sal1	CAGGTCGACTCATGTGGTGATGAAAGTCCAAATCTTC
pCambia-NbNTL9-F-Kpn1	GACGATAAGGGTACCATGATGGCCGTACTTCCTG
pCambia-NbNTL9-R-Xba1	GTCGACTCTAGACTACACACACAGTCTAAAGCATTC
pGD-GFP-CRWN2N-Sal1-R	TGCAGGTCGACTCAGGTTCCAGCAGAAGCAGTAGATC
pCambia-NbNTL9C-F-Kpn1	CGAGCTCGGTACCCGGATGCAGCCTCTAGATTGG
pCambia-TYLCCNV-V2-Flag-F	GAGCTCGGTACCCGGATGTGGGATCCTCTGCTC
pCambia-TYLCCNV-V2-Flag-R	GTCGTCGACTCTAGAGGGCTTCTGTACATCCG
p2YC-CRWN2-F-Pst1	GACGAGCTGTACAAGATGTCTACTCCGCCGAG
p2YC-CRWN2-R-Pst1	GATACGAACGAAAGCTCTGCAGTTATGTGGTGATGAAA GTCCAAATC
p2YN-NbNTL9-F-Pst1	CGACAAGCAGAAGGGAATGATGGCCGTACTTCCTG
p2YN-NbNTL9-R-Pst1	GAACGAAAGCTCTGCAGCTACACACACAGTCTAAAGC ATTCC
p2YN-TYLCCNV-V2-F-Pac1	GAACGATAGTTAATTAAATGTGGGATCCTCTGCTC
p2YN-TYLCCNV-V2-R-Spe1	CACCTCCTCCACTAGTGGGCTTCTGTACATCCGATAC
TRV-NbNTL9-F-BamH1	CCTCCATGGGGATCCTCAATGCAGCCTCTAGATTG
TRV-NbNTL9-R-Xho1	ATGCCCGGGCCTCGAGCTCACTACATGATCCAGAGTCC
NbPR1-pro-F-Sal1-LUC	CATGCCTGCAGGTCGACGACTATTGGAGAAATGTTGTAT TTTGG
NbPR1-pro-R-BamH1-LUC	GTTTTTGGCGTCTTCCATTAAACTTTTTTTAAGGACCAA GAGATG
NbPR1-pro-R-BamH1-LUC △ atg	GTTTTTGGCGTCTTCTAAACTTTTTTTAAGGACCAAGAG ATG
pMBP-NbNTL9-F-Nco1-groesel	GTATTTTCAGGGCGCCATGATGATGGCCGTACTTCCTG
pMBP-NbNTL9-R-Nco1-groesel	GTGACGCCTCGAGTCACTACACACACAGTCTAAAGC
pET28a-his-V2-BamH1-F	CAAATGGGTCGCGGATCCATGTGGGATCCTCTGCTC
pET28a-his-V2-Sal1-R	GCAAGCTTGTCGACTCAGGGCTTCTGTACATCC
pET28a-SUMO-CRWN2-SacI-F	GATCCGAATTCGAGCTCATGTCTACTCCGCCGAG

pET28a-SUMO-CRWN2-XhoI-R	TGGTGGTGCTCGAGTGTGGTGATGAAAGTCCAAATC
TYLCCNV V2(A21P)-F	TTAGGTGTATGTTACCAATTAAGTATTTGCAGTTAGTTG
TYLCCNV V2(A21P)-R	TGCAAATACTTAATTGGTAACATACACCTAAAACCATG
pBridge-Met-GFP-F-KpnI	CTTTAATTTGCGGCCGGTACCTTATTTTTTGCTTTTTCTC TTGAGGTC
pBridge-Met-GFP-R-Pac1	CTATAGGGCGAATTGTTAATTAAAACGCAGAATTTTCGA GTTATTAAAC
pBridge-TYLCCNV-V2-F-Not1	AGAAGAGAAAGGTGGCGGCCGCAATGTGGGATCCTCT GCTC
pBridge-TYLCCNV-V2-R-BgIII	GAGATCAGCCCGAAGATCTTCAGGGCTTCTGTACATCC
**For Quantitative-PCR**	
qPCR-CRWN17-F	CATCCCTCGGTACTGGATGC
qPCR-CRWN17-R	CTGACTTGCCTCCGTGACTT
qPCR-CRWN23-F	CAATACCGAGCGTGAAGCTC
qPCR-CRWN23-R	CATCCTCAAGTTCACTAGCTAGTCGG
qPCR-CRWN13-F	TGATGCTCAGGCTTTGCTCA
qPCR-CRWN13-R	GCTTCCAACTCAGCCTCGAT
qPCR-CRWN17-F	CATCCCTCGGTACTGGATGC
qPCR-CRWN17-R	CTGACTTGCCTCCGTGACTT
qPCR-CRWN02-F	GCCAAGCTTGAAAAAGAGCTTTTTGATTATCAG
qPCR-CRWN02-R	CATATATTATCAACCATACATTGCTTCTTAGAAGCC
qPCR-CRWN2-F	TTCTCCGGCTGGACTTTGAC
qPCR-CRWN2-R	CGCATTGCTTCTCAACACCC
qPCR-TYLCCNV-F	CATGATGTATCGGATGTACAGAAGCCC
qPCR-TYLCCNV-R	GTCCATCCATATCTTCCCAATGACG
qPCR-ToLCNDV-F	CCGAGGAACTGGACTCACAC
qPCR-ToLCNDV-R	TCATACTTGCCGGCCTCTTG
qPCR-TLCYnV-F	ACAGAAGCCCTGATGTTCCG
qPCR-TLCYnV-R	TTCACAGTACCGGTGCTTGG
qPCR-NbNTL9-F	TTGGACCAGTTGAGTCTCGC
qPCR-NbNTL9-R	CCCAGACACCAAGGAAGACC
qPCR-NbPR1-F	ACCTCGTACATTCTCATGGTC
qPCR-NbPR1-R	CGAGTTACGCCAAACCACCTG
qPCR-ChIP-ProPR1- F	ACATGGGCAGTCATCGATGA
qPCR- ChIP-ProPR1- R	ACTCCAGACCAAGTCGTGAT
qPCR- ChIP-ProPR5- F	AGCGAAATCTTTGCGTGTACA
qPCR- ChIP-ProPR5- R	CATGGCCTATGTTGTGTGCG
